# Gravitational waves at the first post-Newtonian order with the Weyssenhoff fluid in Einstein–Cartan theory

**DOI:** 10.1140/epjc/s10052-022-10558-9

**Published:** 2022-07-22

**Authors:** Emmanuele Battista, Vittorio De Falco

**Affiliations:** 1 Department of Physics, University of Vienna, Boltzmanngasse 5, 1090 Vienna, Austria; 2 Scuola Superiore Meridionale, Largo San Marcellino 10, 80138 Napoli, Italy; 3 Istituto Nazionale di Fisica Nucleare, Sezione di Napoli, Complesso Universitario di Monte S. Angelo, Via Cintia Edificio 6, 80126 Napoli, Italy

## Abstract

The generation of gravitational waves from a post-Newtonian source endowed with a quantum spin, modeled by the Weyssenhoff fluid, is investigated in the context of Einstein–Cartan theory at the first post-Newtonian level by resorting to the Blanchet–Damour formalism. After having worked out the basic principles of the hydrodynamics in Einstein–Cartan framework, we study the Weyssenhoff fluid within the post-Newtonian approximation scheme. The complexity of the underlying dynamical equations suggests to employ a discrete description via the point-particle limit, a procedure which permits the analysis of inspiralling spinning compact binaries. We then provide a first application of our results by considering binary neutron star systems.

## Introduction

Nowadays, gravitational-wave (GW) astronomy represents a fundamental mean to investigate gravity at extreme regimes and offers valuable insights into the physics of compact objects [1, 2], as the best GW candidates are represented by black holes (BHs) and neutron stars (NSs) [3]. GWs manifest as perturbations of the spacetime and their theoretical description lays solid roots in general relativity (GR). *The GW theory is intimately intertwined with the two-body problem*. A variety of analytical and numerical techniques has been developed to foretell approximately the dynamics and the corresponding waveforms of compact-object binary systems during their inspiral, plunge, merger, and ringdown stages [4, 5]. The motion and the radiation of post-Newtonian (i.e., slowly moving, weakly stressed, and weakly self-gravitating) isolated sources during their early inspiralling stage can be tackled via the Blanchet–Damour scheme. This framework is built on the pioneering works by Bonnor and collaborators [6–9] and Thorne [10], and exploits two approximation strategies: the multipolar-post-Minkowskian (MPM) and the post-Newtonian (PN) methods [11, 12]. Other two fundamental GW generation formalisms are the Will-Wiseman-Pati approach, which extends the pattern first developed by Epstein and Wagoner [13], and the gravitational self-force (GSF) model. The former reckons with the direct integration of the relaxed Einstein equations (DIRE) and differs from the aforementioned MPM-PN program in the definition of the source multipole moments [14–17]. GSF is based on BH perturbation theory and explores the dynamics and the radiative phenomena of extreme-mass-ratio inspirals [18–21]. The effective-one-body (EOB) framework provides a highly-accurate description of the motion and the gravitational amplitude of coalescing binaries in their late evolution phases [22–27]. A valid support, especially for the demanding task of solving the Einstein field equations in the most extreme regimes, is provided by numerical relativity (NR). Indeed, NR simulations are firmly harnessed to predict waveforms through the merger and the ringdown, and validate the approaches used to study binary systems [28–32]. Furthermore, NR is largely exploited together with several phenomenological patterns (Phenom) to perform the fitting and the parameter estimation of the GW data [33–37].

The state of the art on the conservative dynamics consists in the complete set of the equations of motion of point-particle nonspinning binaries at the fourth post-Newtonian (4PN) order, where the underlying calculations have been undertaken within three different patterns: the Arnowitt-Deser-Misner (ADM) Hamiltonian formulation of GR [38, 39], the Fokker-action approach in harmonic coordinates [40, 41], and the effective-field-theory model [42, 43]; recently, the 5PN and 6PN levels have been worked out modulo a small number of unknowns [44–47]. In addition, this field is currently investigated via new methodologies making use of tools stemming from effective field theory and modern scattering amplitude programs [48–50]. Regarding the GW emission aspects, the 3PN-accurate waveform has been determined [51] and a great deal of efforts is being made in the literature to extend our present knowledge regarding GW templates of inspiralling compact binaries up to 4PN level [52–54]. In the case of spinning binary systems, both the radiative aspects and equations of motion have been worked out with high PN accuracy [55–65].

The huge amount of complementary observational data, triggered by the fast advance of the technological progress, is increasing our capacity to acquire more and more accurate information on gravitational sources and gravity itself. These scientific achievements have motivated us to explore the interplay between quantum and GR effects in GW phenomena via the Einstein–Cartan (EC) theory. Indeed, in a previous paper [66] we have solved, in the context of EC model, the GW generation problem at 1PN level by exploiting the Blanchet–Damour formalism, which permits relating the outgoing radiative gravitational field to the structure and the motion of a spinning PN isolated source. The final solution is encoded by the 1PN-accurate relations for the radiative moments, which are given in the form of well-defined (compact-support) integral expressions over the stress-energy distribution of the matter field. This result can be obtained after a detailed analysis regarding the gauge condition, the coordinates covering the interior and the exterior zone of the source, and the structure of the torsion tensor, which is supposed to admit a vanishing trace. Furthermore, the invariance of the Riemann tensor in the context of the linearized EC framework must be invoked in order to match the internal and the external fields and write the final expressions of the radiative moments involving the new spin contributions. Hereafter, “spin” is intended as the *quantum* (microscopic) angular momentum of elementary particles [78].

In this paper, we apply the abovementioned Blanchet–Damour scheme to a GW source described by the *Weyssenhoff model of an ideal fluid with spin* [67, 68]. This choice is motivated by the fact that it configures as a natural extension of the GR perfect fluid and permits to simplify the demanding calculations framed in EC theory. The hydrodynamical picture of the Weyssenhoff treatment can be obtained by considering first a perfect fluid, and then by assigning to each “fluid element”, which contains a set of microscopic spin configurations, a value of the spin density tensor via an average procedure [69, 70]. The Weyssenhoff fluid has been considered both in cosmological models and astrophysical problems. In the first case, it has been proved that spin interactions in the early stages of the universe could bring about significant results, such as the avoidance of the big-bang singularity, as well as the reproduction of cosmic inflation and dark energy mechanisms [69–72]. In astrophysical settings, the spin may avert the spacetime singularity caused by the gravitational collapse of a star [73–77].

In our analysis, the dynamics of the Weyssenhoff fluid is investigated through the PN method, which permits obtaining a hierarchy of PN mathematical problems to be solved perturbatively order by order starting from the 0PN level. However, this approach still leads to a set of partial and integro-differential dynamical equations, which in general can be tackled through NR. Therefore, in order to obtain analytical results, we resort to the point-particle procedure, which is a valid pattern widely exploited in the literature [17]. In this way, the bodies can be treated as point-like objects and their dynamics is described in terms of ordinary differential equations. This prepares the ground for the analysis of inspiralling spinning compact binaries, which can represent the high-energy astrophysical testbed of our model.

The article is organized as follows: in Sect. 2, we briefly recall the fundamental results of Ref. [66]; in Sect. 3, after having set out the main concepts of the hydrodynamics in EC theory, we deal with the Weyssenhoff model; then, we pass from the continuous description to the discrete picture of the fluid by exploiting the point-particle limit, which is worked out in Sect. 4; in Sect. 5, we apply our theoretical apparatus to NS binaries, and provide a first estimate of EC corrections by studying the ensuing gravitational flux and waveform; eventually, in Sect. 6, we give a summary of the paper and draw the conclusions.

*Notations.* We use metric signature $(-,+,+,+)$. Greek indices take values 0, 1, 2, 3, while the Latin ones 1, 2, 3. The flat metric is indicated by $\eta ^{\alpha \beta }=\eta _{\alpha \beta }=\mathrm{diag}(-1,1,1,1)$. The determinant of the metric $g_{\mu \nu }$ is denoted by *g*. $\epsilon _{kli}$ is the completely antisymmetric Levi-Civita symbol, whose value is 1 if *kli* is an even permutation of 123. Four-vectors are written as $a^\mu = (a^0,\varvec{a})$ and we employ the following notations: $\varvec{a} \cdot \varvec{b} \equiv \delta _{lk}a^l b^k$, $\vert \varvec{a} \vert \equiv a = \left( \varvec{a} \cdot \varvec{a}\right) ^{1/2}$, and $\left( \varvec{a} \times \varvec{b}\right) ^i \equiv \epsilon _{ilk} a^l b^k$. Round (respectively, square) brackets around a pair of indices stands for the usual symmetrization (respectively, antisymmetrization) procedure, i.e., $A_{(ij)}=\frac{1}{2}(A_{ij}+A_{ji})$ (respectively, $A_{[ij]}=\frac{1}{2}(A_{ij}-A_{ji})$).

## First post-Newtonian generation of gravitational waves in Einstein–Cartan theory

We briefly recall the essential information on EC theory and set up the mathematical tools in Sect. 2.1. After that, we introduce the mathematical framework related to the GW generation problem in EC theory at 1PN level (see Sect. 2.2). Then, we outline the procedure, based on the Blanchet–Damour approach, which permits obtaining the approximate solution (see Sect. 2.3). Finally, the general expressions of the asymptotic gravitational waveform and the radiated power are displayed (see Sect. 2.4).

### Einstein–Cartan theory

The EC theory is an extension of GR where both the spin and the mass of matter play a dynamical role. This model is defined on a spacetime $\mathscr {M}$, endowed with a symmetric metric tensor $g_{\alpha \beta }$ and the most general metric-compatible affine connection

1 $$\begin{aligned} \varGamma ^\lambda _{\mu \nu }&=\hat{\varGamma }^{\lambda }_{\mu \nu }-K_{\mu \nu }^{ \lambda }, \end{aligned}$$

where $\hat{\varGamma }^{\lambda }_{\mu \nu }$ denotes the *Christoffel symbols* and $K_{\mu \nu }^{ \lambda }= S_{\nu \mu }^{\lambda }- S^{\lambda }_{\mu \nu } - S_{\mu \nu }^{ \lambda }$ the *contortion tensor*, with $S_{\mu \nu }^{\lambda } \equiv \varGamma ^\lambda _{[\mu \nu ]}$ dubbed the *Cartan torsion tensor*. Hereafter, a hat symbol refers to quantities framed in GR.

Given the matter Lagrangian density, it is possible to introduce the metric energy-momentum tensor $T^{\alpha \beta }$, *the spin angular momentum tensor* $\tau _{\gamma }^{\beta \alpha }$, and the spin energy potential $\mu _{\gamma }^{\beta \alpha }$ [66, 78]. In particular, the tensors $\mu _{\gamma }^{\beta \alpha }$ and $\tau _{\gamma }^{\beta \alpha }$ are related in the following way:

2 $$\begin{aligned} \mu ^{\alpha \beta \gamma }=-\tau ^{\alpha \beta \gamma }+\tau ^{\beta \gamma \alpha }-\tau ^{\gamma \alpha \beta }. \end{aligned}$$

Another fundamental object is the total energy-momentum tensor of matter $\mathbb {T}^{\alpha \beta }$, defined as [78, 79]3where  is the *modified covariant derivative* operator, whose action on a generic tensor field of type (1, 1) is4The EC field equations are ($\chi \equiv 16 \pi G/c^4$) [78]

5a $$\begin{aligned} \hat{G}^{\alpha \beta }&=\frac{\chi }{2}\varTheta ^{\alpha \beta }, \end{aligned}$$

5b $$\begin{aligned} \varTheta ^{\alpha \beta }&\equiv T^{\alpha \beta }+\frac{\chi }{2}\mathcal {S}^{\alpha \beta }, \end{aligned}$$

5c $$\begin{aligned} \mathcal {S}^{\alpha \beta }&\equiv -4\tau ^{\alpha \gamma }{}_[{\delta }\tau ^{\beta \delta }{}_{\gamma }]-2\tau ^{\alpha \gamma \delta }\tau ^\beta {}_{\gamma \delta }+\tau ^{\gamma \delta \alpha }\tau _{\gamma \delta }{}^\beta \nonumber \\&\qquad +\frac{1}{2}g^{\alpha \beta }\left( 4\tau _{\mu }^{\gamma }{}_[{\delta }\;\tau ^{\mu \delta }{}_{\gamma }]+\tau ^{\mu \gamma \delta }\tau _{\mu \gamma \delta }\right) , \end{aligned}$$

where the combined energy-momentum tensor $\varTheta ^{\alpha \beta }$ satisfies

6 $$\begin{aligned} \hat{\nabla }_\beta \varTheta ^{\alpha \beta }=0. \end{aligned}$$

The matter source’s dynamical equations can be obtained via the generalized conservation laws of energy-momentum and angular momentum, which read as, respectively,^1^ [78, 79] 7a7b the Riemann tensor being given by

8 $$\begin{aligned} R^{\mu }_{\nu \rho \sigma }&= \partial _\rho \varGamma ^{\mu }_{\sigma \nu }-\partial _\sigma \varGamma ^{\mu }_{\rho \nu } + \varGamma ^{\mu }_{\rho \alpha } \varGamma ^{\alpha }_{ \sigma \nu }-\varGamma ^{\mu }_{\sigma \alpha } \varGamma ^{\alpha }_{\rho \nu } \nonumber \\&= \hat{R}^{\mu }_{\nu \rho \sigma } + \hat{\nabla }_\sigma K_{\rho \nu }^{\mu }-\hat{\nabla }_\rho K_{\sigma \nu }^{\mu } \nonumber \\&\quad + K_{\rho \alpha }^{\mu }K_{\sigma \nu }^{\alpha }- K_{\sigma \alpha }^{\mu }K_{\rho \nu }^{\alpha }. \end{aligned}$$

### The mathematical problem

The GW generation in EC theory is described by the following well-posed mathematical problem [66]: 
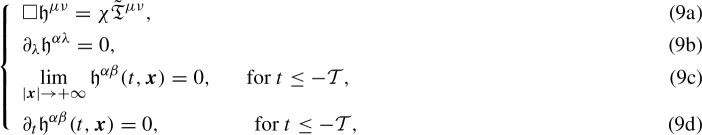
 where $$\mathfrak {h}^{\alpha \beta } \equiv \sqrt{-g}g^{\alpha \beta }-\eta ^{\alpha \beta }$$, $$\Box \equiv \eta ^{\alpha \beta } \partial _\alpha \partial _\beta $$, and

10 $$\begin{aligned} \tilde{\mathfrak {T}}^{\alpha \beta }\equiv \left( -g\right) \, \varTheta ^{\alpha \beta }+\frac{1}{\chi }\varLambda ^{\alpha \beta }, \end{aligned}$$

is the *effective stress-energy pseudotensor* encompassing both the matter fields, described by $\varTheta ^{\alpha \beta }$, and the effective gravitational source term $\varLambda ^{\alpha \beta }=\varLambda ^{\alpha \beta }(\mathfrak {h})$, which includes all the nonlinearities of EC field equations. The harmonic gauge (9b) can be imposed provided we further require [66]

11 $$\begin{aligned} S^{\alpha \mu }_{ \mu }=0. \end{aligned}$$

The gravitational source is supposed to be confined to the region $\varOmega$, defined by

12 $$\begin{aligned} \varOmega =\left\{ \varvec{x}\in \mathbb {R}^3:|\varvec{x}|\le \bar{d}\right\} , \end{aligned}$$

with $\bar{d}$ the typical size of the source. This condition ensures, on the one hand, that $T^{ \alpha \beta }$, $\tau _{\gamma }^{\beta \alpha }$, and $\varTheta ^{\alpha \beta }$ are smooth functions in $\mathbb {R}^4$ having a spatially compact support in $\varOmega$, and, on the other, that the torsion tensor vanishes outside $\varOmega$.

We deal with *PN sources in EC theory*, for which the spatial domain $\mathbb {R}^3$ can be decomposed as $\mathbb {R}^3=\mathfrak {D}_e\cup \mathfrak {D}_i$, where the set $\mathfrak {D}_i$ is the *near zone* and covers entirely the source, while $\mathfrak {D}_e$ encompasses the external weak-field region of the source and is called *exterior zone*. For a PN source these two domains intersect in the *overlapping region* $\mathfrak {D}_o$. Finally, the spatial region where a detector apparatus is located is known as *wave zone*.

### Resolution method

The (approximate) solution of problem (9) can be worked out as follows [66, 81]. First of all, two preliminary steps need to be carried out in the overlapping region $\mathfrak {D}_o$: (1) calculating the multipole (re)expansion of the 1PN series of the inner metric, which can be expressed in terms of the *source multipole moments* $I_L$ and $J_L$^2^; (2) evaluating the 1PN (re)expansion of the MPM external metric, which depends on the *canonical multipole moments* $M_L$ and $S_L$. Eventually, $I_L,J_L$ and $M_L,S_L$ are related to the *radiative moments* $U_L, V_L$ (representing the physical observables in the wave zone) by exploiting the matching procedure. At 1PN order, the relation between $I_L, J_L$ and $U_L, V_L$ is given by (with $l \ge 2$) [66]

13a $$\begin{aligned} U_L(u)&= \overset{(l)}{I}_L(u)+ \mathrm{O}(c^{-3}), \end{aligned}$$

13b $$\begin{aligned} V_L(u)&= \overset{(l)}{J}_L(u) + \mathrm{O}(c^{-2}), \end{aligned}$$

the superscript (*l*) denoting the *l*-th time derivative with respect to the variable *u*, and 14a14b where

15 $$\begin{aligned} \sigma \equiv \dfrac{\varTheta ^{00}+\varTheta ^{kk}}{c^2}, \qquad \sigma _i \equiv \dfrac{\varTheta ^{0i}}{c}, \end{aligned}$$

and  stands for the symmetric-trace-free (STF) projection of $y_L$.

### Asymptotic gravitational waveform and radiated power

Given a set $X^\mu =(cT,\varvec{X})$ of radiative coordinates, the external metric can be put in the so-called *radiative form*, where its coefficients admit an asymptotic expansion in powers of $\mathcal {R}^{-1}$ at future null infinity (i.e., $\mathcal {R} \equiv \vert \varvec{X} \vert \rightarrow \infty$ with $\mathcal {U} \equiv T-\mathcal {R}/c$ and $\varvec{\mathcal {N}} \equiv \varvec{X}/\mathcal {R}$ fixed) [12, 80–83]. The *asymptotic waveform* $\mathscr {H}^\mathrm{TT}_{ij}(X)$ describing the outgoing radiation is defined as the transverse-traceless (TT) projection of the leading $\mathcal {R}^{-1}$ term of such an expansion. At 1PN order, it reads as

16 $$\begin{aligned} \mathscr {H}_{ij}^\mathrm{TT}(X^\mu )&= \dfrac{2G}{c^4 \mathcal {R}} \mathscr {P}_{ijkl}(\varvec{\mathcal {N}}) \Biggr \{ U_{kl}(\mathcal {U}) \nonumber \\&\quad + \dfrac{1}{c} \left[ \dfrac{1}{3} \mathcal {N}_a U_{kla}(\mathcal {U}) +\dfrac{4}{3} \epsilon _{ab(k} V_{l)a}(\mathcal {U}) \mathcal {N}_b \right] \nonumber \\&\quad +\dfrac{1}{c^2} \left[ \dfrac{1}{12}\mathcal {N}_{ab} U_{klab}(\mathcal {U}) \right. \nonumber \\&\quad \left. + \dfrac{1}{2} \epsilon _{ab(k} V_{l)ac}(\mathcal {U}) \mathcal {N}_{bc} \right] + \mathrm{O}(c^{-3}) \Biggr \}, \end{aligned}$$

where

17 $$\begin{aligned} \mathscr {P}_{ijkl} (\varvec{\mathcal {N}})&\equiv \mathscr {P}_{ik}\mathscr {P}_{jl}-\dfrac{1}{2}\mathscr {P}_{ij}\mathscr {P}_{kl}, \nonumber \\ \mathscr {P}_{ij} (\varvec{\mathcal {N}})&\equiv \delta _{ij}-\mathcal {N}_i \mathcal {N}_j, \end{aligned}$$

$\mathscr {P}_{ijkl}(\varvec{\mathcal {N}})$ being the TT projection operator onto the plane orthogonal to $\varvec{\mathcal {N}}$.

Starting from the results contained in Refs. [17, 83, 84], we have proved that the standard GR formula of the *total radiated power* $\mathcal {F}$ (also called, in the astrophysics literature, *total gravitational luminosity or flux of the source*) is valid also in EC theory. Therefore, at 1PN order, the total energy radiated per unit time, expressed as a function of the retarded time $\mathcal {U}$, reads as

18 $$\begin{aligned} \mathcal {F}(\mathcal {U})&= \dfrac{G}{c^5} \Biggl \{\dfrac{1}{5}\overset{(1)}{U}_{ij}(\mathcal {U})\overset{(1)}{U}_{ij}(\mathcal {U}) \nonumber \\&\quad + \dfrac{1}{c^2} \Biggl [ \dfrac{1}{189} \overset{(1)}{U}_{ijk}(\mathcal {U}) \overset{(1)}{U}_{ijk}(\mathcal {U}) \nonumber \\&\quad + \dfrac{16}{45} \overset{(1)}{V}_{ij}(\mathcal {U})\overset{(1)}{V}_{ij}(\mathcal {U}) \Biggr ] + \mathrm{O}(c^{-4}) \Biggr \}. \end{aligned}$$

## The semiclassical spin fluid and its post-Newtonian approximation

Having set out the main aspects of the Blanchet–Damour approach in EC theory, we consider, as a first approach to the description of spin effects inside matter, the class of semiclassical spin fluid models. Before getting to the heart of the discussion, in Sect. 3.1 we first present the main principles of the hydrodynamics in EC theory by making use of the hypothesis (11). In Sect. 3.2, we consider the Weyssenhoff fluid, which represents one of the most common frameworks studied in the literature. Finally, in Sect. 3.3, we investigate the Weyssenhoff fluid dynamics within the PN approximation scheme.

### Hydrodynamics in Einstein–Cartan theory under the hypothesis $S_{\alpha \beta }{}^\beta =0$

In this section, we introduce the basic concepts underlying the hydrodynamics in EC theory.

The analysis of a fluid in EC framework turns out to be more complex than in the GR case, but the assumption (11) entails a great simplification. Indeed, the modified covariant derivative (4) becomes19and the EC covariant divergence of a generic vector $A^\mu$ assumes the same form as in GR, namely

20 $$\begin{aligned} \nabla _\mu A^\mu = \hat{\nabla }_\mu A^\mu -K_{\mu \lambda }{}^\mu A^\lambda = \hat{\nabla }_\mu A^\mu . \end{aligned}$$

The spacetime position of each fluid element is labelled in a system of coordinates by $x^\mu =(ct,\varvec{x})$. The description of the kinematic properties of the fluid can be performed in terms of two fundamental quantities: the timelike four-velocity $u^\mu =\frac{\mathrm{d}x^\mu }{\mathrm{d}\lambda }$ (where $\lambda$ denotes the proper time of an observer comoving with the fluid) and the four-acceleration $a^\mu =u^\nu \nabla _\nu u^\mu$.

It is useful to introduce the projection operator on the spatial hypersurface orthogonal to the timelike four-velocity $u^\mu$

21 $$\begin{aligned} \mathcal {P}^{\mu \nu }= \frac{u^\mu u^\nu }{c^2}+g^{\mu \nu }, \end{aligned}$$

and the *EC substantial derivative* of the vector $\xi ^\mu$ along $u^\mu$ [67]

22 $$\begin{aligned} \dot{\xi }^\mu \equiv u^\nu \nabla _\nu \xi ^\mu . \end{aligned}$$

The latter is the natural extension of the concept used in GR and in classical continuous mechanics, which allows to define the *EC time derivative for densities* [67]

23 $$\begin{aligned} \mathscr {D}\xi ^\mu&\equiv \nabla _\nu \left( u^\nu \xi ^\mu \right) =\dot{\xi }^\mu + \xi ^\mu \hat{\nabla }_\nu u^\nu . \end{aligned}$$

The above derivative operator is applied to objects which are densities (e.g., the spin density tensor, which will be introduced in Sect. 3.2) and has a precise physical meaning. Indeed, its definition relies on the fact that the densities must be not only transported along the fluid worldlines, but it must be also taken into account how the volumes transform during the dynamical evolution^3^ (see Ref. [67], for more details).

The metric stress-energy tensor modeling the relativistic perfect fluid which includes also spin contributions, can be generally written as

24 $$\begin{aligned} T^{\alpha \beta }&=T^{\alpha \beta }_\mathrm{perf}+ \varPhi ^{\alpha \beta }, \nonumber \\ T^{\alpha \beta }_\mathrm{perf}&=e\frac{u^\mu u^\nu }{c^2} + \mathcal {P}^{\mu \nu }P, \end{aligned}$$

where $\varPhi ^{\alpha \beta }$ *is a symmetric tensor containing torsion terms and whose explicit form depends on the chosen spin model*, *P* denotes the isotropic fluid pressure, and

25 $$\begin{aligned} e&= \rho c^2+\varepsilon , \end{aligned}$$

the total energy density, which, in turn, depends on the rest-mass density $\rho$ and the internal energy density $\varepsilon$. We note that *e* can be functionally split in the sum of $\rho c^2$ and $\varepsilon$ due to the perfect fluid hypothesis.

Upon introducing the rest-mass density current

26 $$\begin{aligned} J^\mu =\rho u^\mu , \end{aligned}$$

we can write the conservation equation for the rest mass as (cf. Eq. (20))

27 $$\begin{aligned} \hat{\nabla }_\mu J^\mu =0, \qquad \Leftrightarrow \qquad \mathscr {D}\rho =0. \end{aligned}$$

Furthermore, Eq. (19) permits simplifying the expressions of the canonical stress-energy tensor (3) and of the combined stress-energy tensor (5b), which read as, respectively,

28a $$\begin{aligned} \mathbb {T}^{\alpha \beta }&= T^{\alpha \beta }_\mathrm{perf}+ \varPhi ^{\alpha \beta }-\nabla _\lambda (\mu ^{\alpha \beta \lambda }), \end{aligned}$$

28b $$\begin{aligned} \varTheta ^{\alpha \beta }&= T^{\alpha \beta }_\mathrm{perf}+ \varPhi ^{\alpha \beta }+\frac{\chi }{2}\mathcal {S}^{\alpha \beta }. \end{aligned}$$

Therefore, from Eq. (7a), we have

29 $$\begin{aligned}&\nabla _\nu \left( T^{\mu \nu }_\mathrm{perf}+ \varPhi ^{\mu \nu }-\nabla _\lambda \mu ^{\mu \nu \lambda }\right) =2T^{\lambda \nu }_{\mathrm{perf}}\, S^{\mu }_{\nu \lambda } \nonumber \\&\quad + 2\varPhi _{\lambda \nu }S^{\mu \nu \lambda } -2\nabla _\gamma (\mu _{\lambda \nu }{}^\gamma )S^{\mu \nu \lambda }-\tau _{\nu \rho \sigma }R^{\mu \sigma \nu \rho }. \end{aligned}$$

The dynamics of the perfect fluid can be examined via Eqs. (27) and (29), supplemented by Eq. (7b). In this way, we obtain a system of highly-non-linear differential equations, dubbed *cardinal equations of the hydrodynamics in EC theory*, which can be written as 
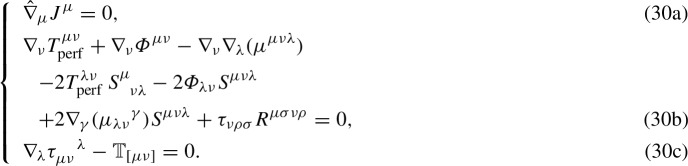


Equation (30b) can be evaluated in the directions orthogonal (by means of the projection operator (21)) and parallel to the fluid four-velocity. The former components give the *Euler equation in EC theory*, while the latter the *energy-balance law*. Furthermore, Eq. (30c) leads to the *rotational equations* of the fluid motion.

The system (30) comprises, in general, 11 independent equations and at most 26 unknowns, which are represented by: the 3 components of the fluid four-velocity (the fourth one is constrained by the normalization condition $u^\mu u_\mu =-c^2$), the rest-mass density $\rho$, the internal energy density $\varepsilon$, the pressure *P*, and (at most) the 20 independent components of the spin angular momentum tensor $\tau _\lambda ^{\ \mu \nu }$. Indeed, the independent components of the torsion tensor $S_\lambda ^{\ \mu \nu }$ have been lowered to 20 by virtue of the hypothesis (11) [66] and hence also $\tau _\lambda ^{\ \mu \nu }$ has (at most) 20 independent components. This is in agreement with the physical content of the EC theory, where the torsion tensor is the geometrical counterpart of the spin of matter, encoded by the tensor $\tau _\lambda ^{\ \mu \nu }$. In the worst case, $S_\lambda ^{\ \mu \nu }$ can be determined once the 20 components of $\tau _\lambda ^{\ \mu \nu }$ are known, but, as we will see in the next section, this number can be drastically reduced by assigning a suitable functional form for $\tau _\lambda ^{\ \mu \nu }$.

It is clear that the system (30) is in general not *closed*, since there is no balance between the number of independent equations and unknowns. However, this not a drawback of the model, which, on the contrary, can reveal a rich dynamical structure. In order to supply the missing equations and correctly characterize the structure of the fluid under investigation, *constitutive equations* must be added to the system (30), e.g., $P=P\left( \rho ,\varepsilon \right)$, $P=P(\varepsilon )$, $\rho =\rho (\varepsilon )$.

A fundamental and general aspect of our approach relies on the possibility of describing the spin effects inside the fluid by exploiting all kinds of (relativistic) spin models. Indeed, the heart of this pattern consists in providing the functional form of $\varPhi ^{\alpha \beta }$ and $\tau ^{\alpha \beta \gamma }$, which is equivalent to assign $T^{\alpha \beta }$ and $\mu ^{\alpha \beta \gamma }$. In general, these two unknowns play a crucial physical and geometrical role in the EC gravity framework. Indeed, we can distinguish the following two classes: (1) *spin-geometry*, represented by either $\tau ^{\alpha \beta \gamma }$ or $\mu ^{\alpha \beta \gamma }$ (skew-symmetric quantities), which rule the torsion field $S_{\mu \nu }{}^\lambda$; (2) *mass-geometry*, described by either $\varPhi ^{\alpha \beta }$ or $T^{\alpha \beta }$ (symmetric quantities), which shape the metric tensor $g_{\mu \nu }$.

### The Weyssenhoff fluid

This section is devoted to the description of the Weyssenhoff fluid. In Sect. 3.2.1, we retrace the historical ideas behind Weyssenhoff approach, since they are useful for its full comprehension. In Sect. 3.2.2, we present the model and the related dynamical equations. We will see that it can be readily analyzed within our general framework put forth in Sect. 3.1. A short digression on the first thermodynamic law is contained in Sect. 3.2.3.

#### Historical introduction to the fluid with spin

The study of matter in its microphysical aspects can be tackled following different strategies framed either in quantum models or from the standpoint of classical vision. Due to the arguments developed in this paper, it is more suitable to follow the latter approach, where the structure of physical systems is derived starting from relativistic theories.

We consider what in the early literature was dubbed *free spin-particle*, namely a material particle endowed with spin and on which no force, apart from the gravitational pull, acts [67, 86, 87]. We pursue the route paved by the “Krakow school” in the years 1937–1947, characterized by the prolific scientific activity of several Polish physicists, like M. Mathisson, J. Lubański, J. Weyssenhoff, and A. Raabe. In 1927, Einstein and Gromer derived the dynamical equations of a free particle from the equations of the gravitational field as the singularities in this field [88]. In 1937, inspired by these ideas, Mathisson and Lubański deduced the equations of motion of a free spin-particle through a variational principle in the case of a linearized gravitational field [86, 87, 89, 90]. This new dynamics exhibits two peculiarities: (1) in the classical limit, it does not reduce to the Newton laws of motion, since it keeps an additional term depending on the internal angular momentum or spin of the particle; (2) the presence of spin generates dynamical equations of the third order. However, Mathisson did not immediately realize that his results were equivalent to those previously published in 1926 by the Russian physicist Frenkel [91]. Indeed, the latter author, after a discussion with Pauli, who showed him a letter by Thomas on the famous “precession factor 1/2” [92], became interested in deriving the equations of motion for a spinning electron, which coincide exactly with those of a free spin-particle only if the terms depending on the electromagnetic field vanish. In 1947, after the Second World War, Weyssenhoff and Raabe proposed a third different method to obtain the equations of a free spin-particle. These authors showed that their results agree with those derived by Frenkel and Mathisson [67]. However, their approach is more rigorous, since it is constructed by exploiting fundamental concepts from continuous mechanics and GR. In addition, the final equations are presented in a much simpler form thanks to the introduction of the linear energy-momentum four-vector [67].

The last approach is the most used in the literature to derive the equations of the incoherent spinning fluid. For this reason, it is also called *Weyssenhoff(-Raabe) fluid*. Having defined $s^{\alpha \beta }$ as the spin density per unit rest-volume, we split it into two three-dimensional vectors

31a $$\begin{aligned} \varvec{s}&=(s^{23},s^{31},s^{12}), \end{aligned}$$

31b $$\begin{aligned} \varvec{q}&=(s^{10},s^{20},s^{30}). \end{aligned}$$

The fundamental hypothesis behind the Weyssenhoff fluid is that the vector $\varvec{q}$ vanishes in the rest system of the fluid, which translates in having the covariant relation [67, 91]

32 $$\begin{aligned} s^{\alpha \beta }u_\beta =0, \end{aligned}$$

since the fluid velocity $u^\alpha =(u^0,\varvec{u})$ has vanishing spatial components when evaluated in the fluid rest frame. This hypothesis, also known in the literature as *Frenkel condition* since it was first employed by Frenkel, arises from the following practical needs: (1) simplifying and closing the set of differential equations underlying the dynamics by matching the numbers of unknowns and equations; (2) letting $\varvec{s}$ be the vector which encodes the real physical degrees of freedom, since $\varvec{q}$ can be gauged away by an appropriate choice of a coordinate system.

Equation (32) can be written in three-dimensional form as

33 $$\begin{aligned}&\varvec{q}=\frac{1}{c}\varvec{u}\times \varvec{s}, \end{aligned}$$

from which it follows

34 $$\begin{aligned} \varvec{q}\cdot \varvec{u}=0. \end{aligned}$$

The vectors $\varvec{s}$ and $\varvec{q}$ have the same transformation properties of the magnetic and the electric fields, respectively. Therefore, the Frenkel condition physically tells us that $s^{\alpha \beta }$ is “purely magnetic”, meaning that its electric component vanishes in the rest-frame coordinate system.

#### The model

We consider the Weyssenhoff semiclassical model of a neutral spinning perfect fluid in the framework of EC theory [68, 69, 78, 93–96].

Following the general approach devised in Sect. 3.1, a specific fluid can be characterized by assigning the spin angular momentum tensor $\tau _{\alpha \beta }{}^\gamma$ and the symmetric tensor $\varPhi ^{\alpha \beta }$. The former, is given by [69]

35 $$\begin{aligned} \tau _{\alpha \beta }{}^\gamma&=s_{\alpha \beta }u^\gamma , \end{aligned}$$

$s_{\alpha \beta }=s_{[\alpha \beta ]}$ being the spin density tensor, which is constrained, due to the hypothesis (11), to satisfy

36 $$\begin{aligned} \tau _{\alpha \beta }{}^\beta = s_{\alpha \beta }\,u^\beta =0, \end{aligned}$$

i.e., the *Frenkel condition* (cf. Eq. (32)). This assumption permits several considerable simplifications. First of all, the torsion and contortion tensors can be written as

37a $$\begin{aligned} S_{\mu \nu }^{\lambda }&= \dfrac{\chi }{2} \left( \tau _{\mu \nu }^{\lambda } \right) , \end{aligned}$$

37b $$\begin{aligned} K_{\mu \nu }^{\alpha }&=\dfrac{\chi }{2} \left( -\tau _{\mu \nu }^{\alpha } +\tau _{\nu \mu }^{\alpha }-\tau ^\alpha _{\mu \nu } \right) , \end{aligned}$$

respectively; furthermore, the EC time derivative of $s_{\mu \nu }$ does not involve contortion terms and reads as (cf. Eq. (23))

38 $$\begin{aligned} \mathscr {D}s_{\mu \nu } = \hat{\nabla }_\lambda \left( s_{\mu \nu } u^\lambda \right) ; \end{aligned}$$

finally, we can write the useful identity

39 $$\begin{aligned} u^\mu \left( \mathscr {D}s_{\mu \nu }\right) = -a^\mu s_{\mu \nu }, \end{aligned}$$

where the acceleration $a^\mu$ is the same as in GR, i.e.,

40 $$\begin{aligned} a^\mu = u^\lambda \hat{\nabla }_\lambda u^\mu =\dot{u}^\mu . \end{aligned}$$

The tensor $\varPhi ^{\alpha \beta }$ of the Weyssenhoff fluid is given by [69]

41 $$\begin{aligned} \varPhi ^{\alpha \beta }&= 2 \left( \dfrac{u_\mu u^\gamma }{c^2}-\delta ^\gamma _\mu \right) \hat{\nabla }_\gamma \left[ s^{\mu (\alpha }u^{\beta )}\right] \nonumber \\&\quad - \chi \left( s^2 u^\alpha u^\beta + c^2 s^{\alpha }_{\lambda } s^{\beta \lambda }\right) , \end{aligned}$$

where $s^2 \equiv s^{\alpha \beta }s_{\alpha \beta }$ is the spin density scalar, satisfying the condition $\mathscr {D}s^2=0$. Therefore, from Eq. (41), jointly with Eqs. (5c), (24), and (35), we have

42 $$\begin{aligned} T^{\alpha \beta }&= e \dfrac{u^\alpha u^\beta }{c^2}+ \mathcal {P}^{\alpha \beta } P + 2 \left( \dfrac{u_\mu u^\gamma }{c^2}-\delta ^\gamma _\mu \right) \hat{\nabla }_\gamma \left[ s^{\mu (\alpha }u^{\beta )}\right] \nonumber \\&\quad - \chi \left( s^2 u^\alpha u^\beta + c^2 s^{\alpha }_{\lambda } s^{\beta \lambda }\right) , \end{aligned}$$

43 $$\begin{aligned} \mathcal {S}^{\alpha \beta }&=2c^2 s^{\alpha }_{\lambda } s^{\beta \lambda } +s^2 u^\alpha u^\beta -\dfrac{1}{2}s^2 c^2 g^{\alpha \beta }, \end{aligned}$$

and hence the explicit expressions of the canonical and the combined energy-momentum tensors are, respectively, (cf. Eq. (28))

44a $$\begin{aligned} \mathbb {T}^{\alpha \beta }&= \left( e u^\alpha - 2 a_\sigma s^{\sigma \alpha } \right) \dfrac{u^\beta }{c^2}+\mathcal {P}^{\alpha \beta } P, \end{aligned}$$

44b $$\begin{aligned} \varTheta ^{\alpha \beta }&= \left( \dfrac{e }{c^2}-\frac{\chi }{2}s^2\right) u^\alpha u^\beta + \mathcal {P}^{\alpha \beta } P -\frac{\chi c^2}{4}s^2g^{\alpha \beta } \nonumber \\&\quad +2\left( \dfrac{u_\mu u^\gamma }{c^2}-\delta ^\gamma _\mu \right) \hat{\nabla }_\gamma \left[ s^{\mu (\alpha }u^{\beta )}\right] . \end{aligned}$$

The general form of the four-momentum density is

45 $$\begin{aligned} p^\alpha = (-p_\mu u^\mu )\dfrac{ u^\alpha }{c^2} + \ell ^\alpha , \end{aligned}$$

where $\ell ^\alpha$ is a spacelike vector orthogonal to $u^\alpha$. An inspection of Eq. (44a) suggests $\ell ^\alpha =-2a_\sigma s^{\sigma \alpha }/c^2$ and hence

46 $$\begin{aligned} p^\alpha = \dfrac{1}{c^2} \left( e u^\alpha - 2 a_\sigma s^{\sigma \alpha }\right) , \end{aligned}$$

upon exploiting the relation $e = -p_\alpha u^\alpha$ [69].

By substituting Eq. (46) in Eq. (44a), we recover the usual form of the canonical energy-momentum tensor for the Weyssenhoff fluid [78], i.e.,

47 $$\begin{aligned} \mathbb {T}^{\alpha \beta } = p^\alpha u^\beta +\mathcal {P}^{\alpha \beta } P. \end{aligned}$$

The internal structure of the fluid is described in terms of the rest-mass density $\rho$ and the internal energy density $\varepsilon$. The total energy density *e* can be written as (cf. Eq. (25))

48 $$\begin{aligned} e&\equiv e(\rho ,\mathfrak {s},s_{\mu \nu })= \rho c^2+\varepsilon (\rho , \mathfrak {s},s_{\mu \nu }), \end{aligned}$$

where $\mathfrak {s}$ denotes the specific entropy (i.e., the entropy per unit mass). The functional dependence of $\varepsilon$ partially resembles that of classical physics, apart from the presence of $s_{\mu \nu }$, which embodies the new contribution due to quantum mechanical effects and is specific of the EC theory.

We suppose that the fluid is *adiabatic* and that the rest mass of the system is conserved. The first hypothesis can be written as [85, 97]

49 $$\begin{aligned} \dot{\mathfrak {s}}=0, \end{aligned}$$

whereas the second one is represented by Eq. (30a).

The translational equations pertaining to the fluid dynamics can be obtained from Eq. (30b), along with Eqs. (2), (35), (37), and (41). As pointed out before, the projection along the fluid four-velocity $u^\mu$ gives the energy-balance law (or energy-conservation equation), which upon exploiting Eq. (30a) reads as

50 $$\begin{aligned} \dot{\varepsilon } + \left( \varepsilon + P \right) \hat{\nabla }_\nu u^\nu =0, \end{aligned}$$

whereas the projection onto the hypersurface orthogonal to $u^\mu$ gives the Euler equation, which after some algebra can be written as

51 $$\begin{aligned} \begin{aligned}&\mathcal {P}^\nu _\mu \partial _\nu P + \dfrac{1}{c^2} \left( P+ e \right) a_\mu - \dfrac{2}{c^2} \hat{\nabla }_\nu \left( u^\nu a^\rho s_{\rho \mu } \right) \\&\quad +\chi a^\lambda s_{\lambda \rho } s_{\mu }^{\rho }= - s_{\nu \rho } u^\sigma R_{\mu \sigma }^{\nu \rho }. \end{aligned} \end{aligned}$$

We note that, for $s_{\mu \nu }=0$, Eq. (51) reduces to the Euler equation of GR [17, 85].

Bearing in mind Eqs (30c), (35), (37), and (44a), we find that the rotational fluid motion is ruled by

52 $$\begin{aligned} \mathscr {D}s_{\mu \nu }&= \dfrac{a^\sigma }{c^2} \left( u_\mu s_{ \sigma \nu }- u_\nu s_{ \sigma \mu } \right) , \end{aligned}$$

where we have exploited Eq. (46). Since the tensor $s_{\mu \nu }$ has in general six independent components, the Frenkel condition (36) is crucial to correctly account for the three true dynamical degrees of freedom associated with the spin of a particle. For this reason, Eq. (52) gives rise to three independent equations.

As discussed in Sect. 3.1, the system of the cardinal hydrodynamic equations can be closed once the needed constitutive equations are provided. Therefore, the full set of differential equations governing the Weyssenhoff fluid dynamics is (see Eqs. (27) and (49)–(52)) 
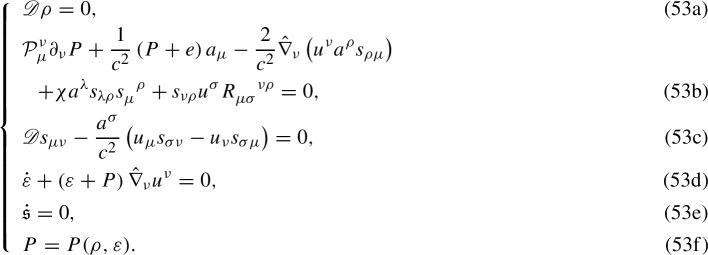


The above system contains ten independent equations and exactly ten unknowns, i.e., the three components of the fluid velocity $u^\mu$, the rest-mass density $\rho$, the internal energy density $\varepsilon$, the specific entropy $\mathfrak {s}$, the isotropic pressure *P*, and the three independent components of $s_{\mu \nu }$. It is also worth noting that it explicitly includes an equation describing the behaviour of $\mathfrak {s}$ (cf. Eq. (53e)). This is due to the fact that, unlike GR [17, 85], in EC theory Eqs. (53a) and (53d) do not imply that the fluid evolves adiabatically (see Sect. 3.2.3).

In conclusion, the closed self-consistent system of gravity-matter equations is represented by EC field equations (5) supplemented by the set (53).

#### The first thermodynamic law

In the study of the hydrodynamics, it is important to investigate the implications of the energy-conservation equation (53d) at thermodynamic level [69, 85]. However, we note that this analysis does not provide additional constraining equations to the system (53), but it allows to better understand how the fluid behaves in EC theory.

The *first thermodynamic law* for a spinning fluid can be written as [69, 93, 96, 98]

54 $$\begin{aligned} \mathrm{d} \varPi =\dfrac{P}{\rho ^2} \mathrm{d}\rho + \theta \mathrm{d}\mathfrak {s} + \dfrac{1}{2} \dfrac{\omega _{\mu \nu }}{\rho } \mathrm{d}s^{\mu \nu }, \end{aligned}$$

where

55 $$\begin{aligned} \varPi \equiv \varPi (\rho ,\mathfrak {s},s_{\mu \nu }) \equiv \dfrac{\varepsilon }{\rho }, \end{aligned}$$

is the *specific internal energy*, and

56a $$\begin{aligned} \left( \dfrac{\partial \varPi }{\partial \rho }\right) _{\mathfrak {s},s^{\mu \nu }}&\equiv \dfrac{P}{\rho ^2}, \end{aligned}$$

56b $$\begin{aligned} \left( \dfrac{\partial \varPi }{\partial \mathfrak {s}}\right) _{\rho ,s^{\mu \nu }}&\equiv \theta , \end{aligned}$$

56c $$\begin{aligned} \left( \dfrac{\partial \varPi }{\partial s^{\mu \nu }}\right) _{\rho ,\mathfrak {s}}&\equiv \dfrac{1}{2} \dfrac{\omega _{\mu \nu }}{\rho }, \end{aligned}$$

$\theta$ being the temperature and $\omega _{\mu \nu }$ the thermodynamic variable conjugated to $s^{\mu \nu }$ coinciding with the microscopic angular velocity of the fluid.

Starting from the continuity Eq. (53a) and the energy-balance law (53d), we obtain

57 $$\begin{aligned} \dot{\varPi } - \dfrac{P}{\rho ^2} \dot{\rho }=0, \end{aligned}$$

which, once compared with Eq. (54), gives

58 $$\begin{aligned} \theta \dot{\mathfrak {s}} + \dfrac{1}{2} \dfrac{\omega ^{\mu \nu }}{\rho } \dot{s}_{\mu \nu }=0. \end{aligned}$$

Bearing in mind Eq. (53e), it follows that

59 $$\begin{aligned} \omega ^{\mu \nu } \dot{s}_{\mu \nu }=0. \end{aligned}$$

At this point, some remarks are in order. First of all, as pointed out before, it is known that in GR a perfect fluid evolves adiabatically [17, 85]. This result can be proved by following the same procedure as the one we have adopted in this section, i.e., by exploiting the fluid continuity equation jointly with the energy-balance law. On the contrary, in EC theory the same calculations do not imply the adiabatic-flow hypothesis as long as the spin contribution in Eq. (58) does not vanish. This shows that in EC hydrodynamics the adiabatic condition must be explicitly imposed via Eq. (53e), which in fact has been employed to obtain the final relation (59).

### Post-Newtonian analysis of the Weyssenhoff fluid dynamics

In this section, we perform the PN investigation of the Weyssenhoff fluid dynamics, which is useful both for the calculations carried out in Sect. 4 and, in general, for several other applications. In Sect. 3.3.1, we outline the PN computational scheme. In Sect. 3.3.2, we present the PN expansion of some basic quantities which are then exploited to compute the 0PN and 1PN orders of Eq. (53) in Sects. 3.3.3 and 3.3.4, respectively.

#### The computational program

The EC hydrodynamics, although equipped with the simplifying constraint $S_{\alpha \beta }{}^\beta =0$, gives rise to a set of coupled nonlinear differential equations (cf. Eq. (30)). Also in the more specific case, where we consider the Weyssenhoff fluid endowed with the Frenkel condition (36), the nonlinear nature and the differential structure still endure (cf. Eq. (53)), and we are, in general, not able to solve the mathematical problem exactly. An analytical solution may be determined by imposing symmetry requirements, like time independence and spatial isotropy (as it occurs, for example, in GR for the Schwarzschild metric). However, this approach reveals to be very limited in its range of applications, where time variability and spatial anisotropies occur frequently.

Since the EC gravity framework is geometrically more tangled than GR theory, our goal is therefore not to search for further exact solutions, but rather to take advantage of a *comprehensive and solid approximation method*, which goes beyond the symmetries and the particular functional form of the starting problem. The mathematical formulation of the GW generation problem naturally suggests us to resort to the *perturbation theory* (see Refs. [99–101], for further details and examples). This approach permits to determine a solution under the form of a power series of a small parameter $\epsilon$ (represented, in our case, by $\epsilon =1/c$), where expressions of higher powers of $\epsilon$ become smaller as the order increases. The *approximate perturbed solution* is obtained by truncating such a series at a certain established order, suggested either by the observational sensitivity of the phenomenon under investigation or, from a theoretical point of view, by the computational complexity (i.e., gradually increasing the PN order calculations).

The strategy to build up the solution via the PN formalism relies on expanding all the involved quantities, and then extracting the coefficients of the expansions which are useful to construct the differential equations at each PN level. In this way, we obtain a series of *PN problems*, where the lowest order permits obtaining the classical equations with eventual spin corrections, and the higher terms provide the successive perturbative corrections. The method is therefore iteratively based on first determining (either analytically or numerically) the solution of the 0PN problem, and then proceeding *order by order* to solve (either analytically or numerically) the successive PN problems. Indeed, to compute the *N*th PN order, we need to use the parameters determined in the previous iterations. Finally, gathering all these quantities together we can provide an approximate solution to the original problem up to the desired PN level.

It is important to remark that we apply this computational program in the derivation of the 0PN and 1PN orders of Eq. (53) without solving them. As we will see, the obtained results will be useful in Sect. 4.

#### Preliminary: expansions of basic quantities

Given the harmonic coordinates $x^\mu = (ct,\varvec{x})$ (cf. Eq. (9b)), the 1PN series of the metric tensor, expressed in terms of the retarded potentials *V* and $V_i$, reads as [66]

60a $$\begin{aligned} g_{00}&= - \mathrm{e}^{-2V/c^2} + \mathrm{O}(c^{-6}), \end{aligned}$$

60b $$\begin{aligned} g_{0i}&= -\dfrac{4}{c^3} V_i + \mathrm{O}(c^{-5}), \end{aligned}$$

60c $$\begin{aligned} g_{ij}&= \delta _{ij} \left( 1 + \dfrac{2}{c^2}V\right) + \mathrm{O}(c^{-4}), \end{aligned}$$

where (cf. Eq. (15))

61a $$\begin{aligned} V\left( t,\varvec{x} \right)&= G \int \dfrac{\mathrm{d}^3 \varvec{y}}{\vert \varvec{x}- \varvec{y}\vert } \sigma \left( t- \vert \varvec{x}- \varvec{y}\vert /c, \varvec{y}\right) , \end{aligned}$$

61b $$\begin{aligned} V_i \left( t,\varvec{x} \right)&= G \int \dfrac{\mathrm{d}^3 \varvec{y} }{\vert \varvec{x}- \varvec{y}\vert } \sigma _i \left( t- \vert \varvec{x}- \varvec{y}\vert /c. \varvec{y}\right) . \end{aligned}$$

The expansion of Eq. (61) for small retardation effects gives

62a $$\begin{aligned} V&= U + \dfrac{1}{2c^2} \partial ^2_t X + \mathrm{O}\left( c^{-3}\right) , \end{aligned}$$

62b $$\begin{aligned} V_i&= U_i + \mathrm{O}\left( c^{-2}\right) , \end{aligned}$$

the potentials *U*, $U_i$, and the superpotential *X* being given by

63a $$\begin{aligned} U\left( t,\varvec{x} \right)&= G \int \dfrac{\mathrm{d}^3 \varvec{y}}{\vert \varvec{x}- \varvec{y}\vert } \sigma \left( t, \varvec{y}\right) , \end{aligned}$$

63b $$\begin{aligned} U_i\left( t,\varvec{x} \right)&= G \int \dfrac{\mathrm{d}^3 \varvec{y}}{\vert \varvec{x}- \varvec{y}\vert } \sigma _i \left( t, \varvec{y}\right) , \end{aligned}$$

63c $$\begin{aligned} X(t,\varvec{x})&= G \int \mathrm{d}^3 \varvec{y}\, \vert \varvec{x}- \varvec{y}\vert \, \sigma \left( t, \varvec{y}\right) , \end{aligned}$$

respectively. It should be noted that the term of order $c^{-3}$ in Eq. (62a) is a function of time only.

Stating from Eq. (60), one easily obtains the PN structure of the Christoffel symbols (see Ref. [99], for details.)

The spin angular momentum tensor $\tau _{\lambda }^{\mu \nu }$ admits, in general, the following PN series [66]:

64 $$\begin{aligned} \tau _{0}^{i 0}&={}^{(0)}\tau _{0}^{i 0}+ {}^{(2)}\tau _{0}^{i 0} + \mathrm{O}\left( c^{-2}\right) , \nonumber \\ \tau _{0}^{i j}&={}^{(1)}\tau _{0}^{i j}+ {}^{(3)}\tau _{0}^{i j} + \mathrm{O}\left( c^{-3}\right) , \nonumber \\ \tau _{i}^{ j 0}&={}^{(1)}\tau _{i}^{j 0}+ {}^{(3)}\tau _{i}^{j 0} + \mathrm{O}\left( c^{-3}\right) , \nonumber \\ \tau _{i}^{ j k}&={}^{(2)}\tau _{i}^{j k}+ {}^{(4)}\tau _{i}^{j k} + \mathrm{O}\left( c^{-4}\right) , \end{aligned}$$

where ${}^{(n)}\tau _{\lambda }^{\mu \nu } \sim \dfrac{\bar{M}}{\bar{d}^2} \dfrac{\bar{v}^n}{c^{n-2}}$ ($\bar{v}$ and $\bar{M}$ being some typical internal velocity and mass of the source, respectively). Therefore, the tensor (5c) has the PN structure

65 $$\begin{aligned}&\mathcal {S}^{00}={}^{(0)}\mathcal {S}^{00} + {}^{(1)}\mathcal {S}^{00} +{}^{(2)}\mathcal {S}^{00} + \mathrm{O}\left( c^{-2}\right) , \nonumber \\&\mathcal {S}^{0i}={}^{(0.5)}\mathcal {S}^{0i} + {}^{(1.5)}\mathcal {S}^{0i} +{}^{(2.5)}\mathcal {S}^{0i} + \mathrm{O}\left( c^{-3}\right) , \nonumber \\&\mathcal {S}^{ij}={}^{(0)}\mathcal {S}^{ij} + {}^{(1)}\mathcal {S}^{ij} +{}^{(2)}\mathcal {S}^{ij}+ \mathrm{O}\left( c^{-2}\right) , \end{aligned}$$

with $${}^{(n)}\mathcal {S}^{\mu \nu } \sim \dfrac{\bar{M}^2}{\bar{d}^4} \dfrac{\bar{v}^{2n}}{c^{2n-4}}$$.

Starting from Eq. (64) along with the PN expression of the Christoffel symbols, it is possible to derive the PN form of the torsion and contortion tensors (37), the connection coefficients (1), and the Riemann tensor (8). The components of the the Riemann tensor needed at 1PN level are reported in Appendix A.

Bearing in mind the definition of the spin angular momentum of a test particle in EC theory (see Eq. (40) of Ref. [66]), for the spin density tensor we find

66 $$\begin{aligned} \begin{aligned} s_{0i}&= {}^{(0)}s_{0i} +{}^{(2)}s_{0i} +\mathrm{O}(c^{-3}), \\ s_{ij}&= {}^{(1)}s_{ij} + {}^{(3)}s_{ij} +\mathrm{O}(c^{-4}), \end{aligned} \end{aligned}$$

where ${}^{(n)}s_{\mu \nu }$ indicates a factor going like $\dfrac{\bar{M} \bar{v}^n}{\bar{d}^2 c^{n-1}}$. If we write the fluid four-velocity equivalently as

67 $$\begin{aligned} u^\mu = \gamma \left( c,\varvec{v}\right) , \end{aligned}$$

(with $\gamma \equiv \tfrac{u^0}{c}$ and $\varvec{v} \equiv \tfrac{\mathrm{d}\varvec{x}}{\mathrm{d}t}$) its PN expansion can be easily constructed by means of Eq. (60) and the normalization condition. Therefore, the Frenkel condition (36) leads to

68a $$\begin{aligned}&{}^{(0)}s_{i0} \left( {}^{(1)}u^i + {}^{(3)}u^i\right) \nonumber \\&\quad + {}^{(2)}s_{i0} \, {}^{(1)}u^i + \mathrm{O}(c^{-3})=0, \end{aligned}$$

68b $$\begin{aligned}&{}^{(0)}s_{j0} \left( {}^{(0)}u^0 + {}^{(2)}u^0\right) + {}^{(2)}s_{j0}\,{}^{(0)}u^0 \nonumber \\&\quad + {}^{(1)}s_{ji} \,{}^{(1)}u^i+ \mathrm{O}(c^{-2})=0, \end{aligned}$$

where ${}^{(n)}u^\mu$ denotes a term of order $\dfrac{\bar{v}^n}{c^{n-1}}$. We note that Eq. (68a) results from a linear combination of Eq. (68b) with coefficients $v^j/c$ (recalling that ${}^{(n)}u^j=\tfrac{{}^{(n-1)}u^0}{c} v^j$ with $n=1,3$). This implies that the latter gives rise to three independent relations which can be used to gauge away the components $s_{0i}$ of the spin density tensor at different PN orders in the equations of motion (cf. Sect. 3.2.1 and the discussion below Eq. (52)). Indeed, from Eq. (68b) we obtain to leading order and to next-to-leading order, respectively,

69a $$\begin{aligned}&{}^{(0)}s_{0i} =\mathrm{O}(c^{-1}), \end{aligned}$$

69b $$\begin{aligned}&{}^{(0)}s_{0i} \left[ 1+ \frac{1}{c^2}\left( V+\frac{v^2}{2}\right) \right] +{}^{(2)}s_{0i}={}^{(1)}s_{ik} \dfrac{v^k}{c}+\mathrm{O}(c^{-3}), \end{aligned}$$

or, equivalently,

70a $$\begin{aligned} {}^{(0)}s_{0i}&=0, \end{aligned}$$

70b $$\begin{aligned} {}^{(2)}s_{0i}&=\frac{{}^{(1)}s_{ik}v^k}{c}. \end{aligned}$$

From the above equations it is clear that no components $s_{0i}$ are present at leading order, whereas they can be written in terms of ${}^{(1)}s_{ik}$ at next-to-leading order. Furthermore, we see that Eq. (68a) simply states that the three-vectors $s_{0i}$ and $u^i$ are perpendicular, but gives no information about the form of $s_{0i}$. On the contrary, Eq. (68b) allows to write explicitly, at each PN level, an expression for $s_{0i}$ which turns out to be both orthogonal to $u^i$ and vanishing in the fluid rest frame.

For our forthcoming calculations, it is useful to express Eq. (53a) in an equivalent form. Indeed, bearing in mind Eq. (67), and upon introducing the rescaled mass density $\rho ^{\star } \equiv \gamma \sqrt{-g} \rho$ [17], Eq. (53a) yields the (exact) equation

71 $$\begin{aligned} \partial _t \rho ^{\star } + \partial _j \left( \rho ^{\star } v^j\right) =0, \end{aligned}$$

the relation between $\rho ^{\star }$ and the proper rest-mass density $\rho$ being

72 $$\begin{aligned} \rho ^\star = \rho \left[ 1+\dfrac{1}{c^2}\left( \dfrac{1}{2} v^2 +3V\right) + \mathrm{O}(c^{-4}) \right] . \end{aligned}$$

The PN expansion of the first thermodynamic law (54) reads as (cf. Eq. (55))

73 $$\begin{aligned} \rho ^\star \dfrac{\mathrm{d}\varepsilon }{\mathrm{d}t}&=\left( \varepsilon +P\right) \dfrac{\mathrm{d}\rho ^\star }{\mathrm{d}t} + \left( \rho ^\star \right) ^2 \theta \dfrac{\mathrm{d}\mathfrak {s}}{\mathrm{d}t} + \dfrac{1}{2} \rho ^\star \omega _{\alpha \beta }\dfrac{\mathrm{d}s^{\alpha \beta }}{\mathrm{d}t} \nonumber \\&\quad + \mathrm{O}\left( c^{-2}\right) , \end{aligned}$$

where

74 $$\begin{aligned} \dfrac{\mathrm{d}}{\mathrm{d}t} f(t,\varvec{x})= \partial _t f + v^k \partial _k f, \end{aligned}$$

and we have exploited Eq. (72), the relations $\omega _{0i} = \mathrm{O}\left( c^{-1}\right)$ and $\omega _{ij} = \mathrm{O}\left( c^0\right)$ (see Refs. [69, 109] for details), and we have supposed that both $\theta$ and $\mathfrak {s}$ are $\mathrm{O}(c^0)$ quantities.

#### Equations of motion: 0PN expansion

The results contained in Sect. 3.3.2 allow us to study the hydrodynamic equations (53) within the PN formalism. In our investigation, the rest-mass conservation Eq. (53a) will be replaced by its equivalent expression (71) and will not be expanded. Furthermore, by means of Eq. (67), Eq. (53e) assumes the exact form

75 $$\begin{aligned} \dfrac{\mathrm{d}\mathfrak {s}}{\mathrm{d}t}=0, \end{aligned}$$

and the constitutive Eq. (53f) will be simply written in terms of $\rho ^\star$ (cf. Eq. (72)). Therefore, by computing the 0PN expansion of the remaining equations of the system (53) and by exploiting the Frenkel condition (70a), we end up, after a lengthy calculation, with the following system pertaining to the dynamics of a Weyssenhoff fluid with 0PN accuracy:
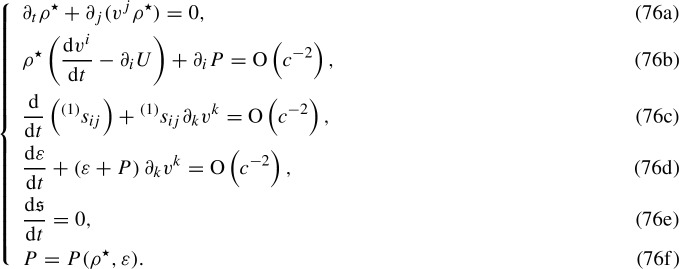


Equation (76b) originates from the 0PN expansion of Eq. (53b) and represents the Euler equation of the Newtonian theory. Equation (76c) is the leading-order piece of Eq. (53c). *At this level, only the derivative* $\mathscr {D}s_{i j}$ *gives a contribution and we end up with a homogeneous continuity equation for* ${}^{(1)}s_{ij}$. Finally, Eq. (76d) is the 0PN-accurate energy-conservation Eq. (53d).

#### Equations of motion: 1PN expansion

In this section, we present the 1PN expressions of Eqs. (53b)–(53d).

The 1PN Euler equation (53b) reads as

77 $$\begin{aligned}&\rho ^\star \left( \dfrac{\mathrm{d}v^i}{\mathrm{d}t} - \partial _i U \right) + \partial _i P +\dfrac{v^i}{c^2}\dfrac{\mathrm{d}P}{\mathrm{d}t} -\dfrac{1}{2c^2} \rho ^\star \partial _i \partial ^2_t X \nonumber \\&\quad +\dfrac{1}{c^2}\left( \dfrac{\mathrm{d}v^i}{\mathrm{dt}} -\partial _i U\right) \left[ P+\varepsilon -\rho ^\star \left( \frac{v^2}{2}+3U\right) \right] \nonumber \\&\quad +\dfrac{\rho ^\star }{c^2}\left\{ \left( \frac{v^2}{2}+3U\right) \dfrac{\mathrm{d}v^i}{\mathrm{d}t}+ 2 v^i \dfrac{\mathrm{d}U}{\mathrm{d}t} - 4 \dfrac{\mathrm{d}U_i}{\mathrm{d}t} \right. \nonumber \\&\quad + \left. \dfrac{\mathrm{d}}{\mathrm{d}t}\left[ v^i\left( U+\dfrac{v^2}{2} \right) \right] -2 v^2 \partial _i U + 4 v^l \partial _i U_l \right\} \nonumber \\&\quad -\dfrac{2}{c^2}\left\{ \dfrac{\mathrm{d}}{\mathrm{d}t}\left[ {}^{(1)}s_{ki}\left( \dfrac{\mathrm{d}v^k}{\mathrm{dt}} -\partial _k U\right) \right] \right. \nonumber \\&\quad \left. +\left( \dfrac{\mathrm{d}v^k}{\mathrm{dt}} -\partial _k U\right) {}^{(1)}s_{ki}\partial _lv^l\right\} +\dfrac{2}{c^2}\,{}^{(1)}s_{jk}\Biggr [-v^k\partial _i\partial _j U \nonumber \\&\quad +v^l\left( \delta _{i[k}\partial _{j]}\partial _l+\delta _{l[j}\partial _{k]}\partial _i\right) U+\frac{\chi \,c^4}{2}\partial _{[k} {}^{(1)}s_{i|j]}\nonumber \\&\quad +2\partial _i \partial _{[j} U_{k]}+\delta _{i[k}\partial _{j]}\partial _t U\Biggr ]=\mathrm{O}\left( c^{-4}\right) , \end{aligned}$$

whereas at 1PN order the spin Eq. (53c) becomes

78 $$\begin{aligned}&\dfrac{\mathrm{d}}{\mathrm{d}t} \left( {}^{(1)}s_{ij}\right) +{}^{(1)}s_{ij}\partial _k v^k \nonumber \\&\quad +\dfrac{\mathrm{d}}{\mathrm{d}t} \left[ \dfrac{{}^{(1)}s_{ij}}{c^2} \left( \dfrac{v^2}{2}+U\right) + {}^{(3)}s_{ij}\right] \nonumber \\&\quad + \left[ \dfrac{{}^{(1)}s_{ij}}{c^2} \left( \dfrac{v^2}{2}+U\right) +{}^{(3)}s_{ij}\right] \partial _k v^k \nonumber \\&\quad + \dfrac{1}{c^2} \dfrac{\mathrm{d}v^k}{\mathrm{d}t} \left[ v^j \, {}^{(1)}s_{ki}-v^i \, {}^{(1)}s_{kj} \right] \nonumber \\&\quad +\dfrac{2}{c^2} \Biggl [ {}^{(1)}s_{ki} \left( \partial _k U_j -\partial _j U_k + v^k \partial _j U -\dfrac{v^j}{2} \partial _k U\right) \nonumber \\&\quad - {}^{(1)}s_{kj} \left( \partial _k U_i -\partial _i U_k + v^k \partial _i U -\dfrac{v^i}{2} \partial _k U\right) \Biggr ] \nonumber \\&\quad + \dfrac{1}{c^2} \left( v^i \,{}^{(1)}s_{lj} \partial _l U - v^j \,{}^{(1)}s_{li} \partial _l U \right) =\mathrm{O}\left( c^{-4}\right) . \end{aligned}$$

In deriving Eqs. (77) and (78), we have exploited Eqs. (62) and (70).

The energy-balance law (53d) yields, at 1PN order,

79 $$\begin{aligned}&\dfrac{\mathrm{d}\varepsilon }{\mathrm{d}t} +\left( \varepsilon +P\right) \partial _j v^j +\dfrac{1}{c^2} \left( \dfrac{v^2}{2}+U\right) \dfrac{\mathrm{d}\varepsilon }{\mathrm{d}t} \nonumber \\&\qquad +\dfrac{\left( \varepsilon + P\right) }{c^2}\Biggl [ \dfrac{\mathrm{d}}{\mathrm{d}t} \left( \dfrac{v^2}{2}+3U\right) + \left( \dfrac{v^2}{2}+U\right) \partial _k v^k \Biggr ] \nonumber \\&\quad =\mathrm{O}\left( c^{-4}\right) , \end{aligned}$$

where we have exploited Eq. (62a).

Therefore, the Weyssenhoff fluid is described, at 1PN order, by the following system: 
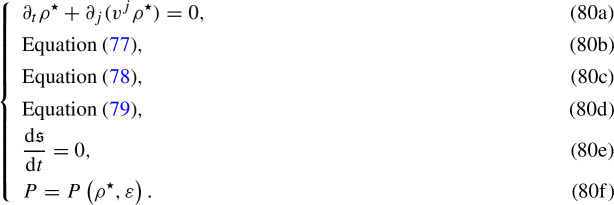


## The point-particle limit of the Weyssenhoff fluid

The final outcome of the PN treatment contained in Sect. 3 is represented by Eqs. (76) and (80), which completely determine the behaviour of the Weyssenhoff fluid at 0PN and 1PN level, respectively. In general, these two sets comprise both partial and integro-differential equations, whose resolution, either analytically or via numerical means, turns out to be highly challenging. This makes, as a consequence, the evaluation of the radiative moments parametrizing the asymptotic waveform and the radiated power (cf. Sect. 2.4) rather demanding.

A widely used approach in the literature which offers a way around this issue consists in employing the so-called *point-particle procedure*, where the fluid configuration is supposed to be described by a collection of separated point-like components, usually refereed to as “bodies”. Within this pattern, the equations underlying the fluid dynamics become less complex as they are turned into *ordinary differential equations*. For these reasons, in Sect. 4.1, we introduce the point-particle procedure and work out the 1PN-accurate expressions of the radiative moments. As we will see, the results of Sect. 3 will be crucial, since they allow us to go from the fine-grained description to the coarse-grained picture of the Weyssenhoff fluid. Finally, in Sect. 4.2, we deal with the special case of binary systems, which are known to be the main candidates of GW events in high-energy astrophysics.

### The point-particle procedure

The evaluation of the 1PN-accurate total power of emission (18) requires the knowledge of the 1PN radiative mass quadrupole moment $U_{ij}$ as well as the 0PN mass octupole $U_{ijk}$ and current quadrupole $V_{ij}$; on the other hand, the 1PN asymptotic waveform (16) can be worked out once the 0PN mass $2^4$-pole $U_{ijkl}$ and current octupole $V_{ijk}$ are also known. Having solved the 1PN GW generation problem [66], we know that these are related to the source multipole moments via Eqs. (13) and (14). Therefore, in order to compute the point-particle limit of the required radiative moments, we first need to determine their fine-grained expression, i.e., the form assumed in the case of a continuous distribution of matter represented by the Weyssenhoff fluid, see Sect. 4.1.1. Then, after some premises outlined in Sects. 4.1.2 and 4.1.3, the point-particle limit is performed in Sect. 4.1.4.

#### The fine-grained form of the radiative moments

In this section, we calculate the fine-grained expression of the radiative moments occurring in the total power of emission and the wave amplitude for the specific case of the Weyssenhoff fluid. This entails, first of all, the computation of the PN expansion of metric stress-energy tensor (42) and the tensor (43). With the help of the results of Sect. 3.3.2, the former yields

81a $$\begin{aligned} {}^{(0)} T^{00}&= \rho c^2, \end{aligned}$$

81b $$\begin{aligned} {}^{(2)} T^{00}&= \varepsilon +\rho (2U+v^2)-2\partial _i\left( {}^{(1)}s_{ik} v^k\right) , \end{aligned}$$

81c $$\begin{aligned} {}^{(1)} T^{0i}&= c\left[ \rho v^i-\partial _k\left( {}^{(1)}s_{ki}\right) \right] , \end{aligned}$$

81d $$\begin{aligned} {}^{(2)} T^{ij}&= \rho v^iv^j+\delta ^{ij}P-2\partial _k\left( {}^{(1)}s_{k(i}v^{j)}\right) , \end{aligned}$$

whereas the latter

82 $$\begin{aligned} {}^{(0)}\mathcal {S}^{00}={}^{(0.5)}\mathcal {S}^{0i}={}^{(0)}\mathcal {S}^{ij}=0, \end{aligned}$$

where we have exploited Eqs. (62a) and (70), and ${}^{(n)}{T}{_{\mu \nu }}$ stands for the contributions in ${T}{_{\mu \nu }}$ of order $\dfrac{\bar{M} \bar{v}^n}{\bar{d}^3 c^{n-2}}$.

By means of Eqs. (72), (81), and (82), we have

83a $$\begin{aligned} \sigma&=\rho ^{\star \star } + \rho _{\mathrm{v}} -\dfrac{4}{c^2}\partial _k\left( s_{kl}v^l\right) + \mathrm{O}\left( c^{-4}\right) , \end{aligned}$$

83b $$\begin{aligned} \sigma _i&=\rho ^\star v^i-\partial _k s_{ki} +\mathrm{O}\left( c^{-2}\right) , \end{aligned}$$

83c $$\begin{aligned} \sigma _{ii}&=\rho ^\star \left( v^2+ \dfrac{3P}{\rho ^\star }\right) -2\partial _k\left( s_{kl}v^l\right) + \mathrm{O}\left( c^{-2}\right) , \end{aligned}$$

where $\sigma$ and $\sigma _i$ have been defined in Eq. (15), while $\sigma _{ij} \equiv \varTheta ^{ij}$ [66], and, inspired by Ref. [102],

84 $$\begin{aligned} \rho ^{\star \star }&\equiv \rho ^\star \left[ 1+\dfrac{1}{c^2} \left( \dfrac{v^2}{2}+ \varPi -\dfrac{U}{2}\right) \right] , \end{aligned}$$

85 $$\begin{aligned} \rho _\mathrm{v}&\equiv \dfrac{1}{c^2} \rho ^\star \left( v^2 -\dfrac{U}{2}+ \dfrac{3P}{\rho ^\star }\right) , \end{aligned}$$

with (see Eq. (63a))

86 $$\begin{aligned} U\left( t,\varvec{x} \right)&= G \int \dfrac{\mathrm{d}^3 \varvec{x}^\prime }{\vert \varvec{x}- \varvec{x}^\prime \vert } \rho ^\star \left( t,\varvec{x}^\prime \right) +\mathrm{O}\left( c^{-2}\right) . \end{aligned}$$

It should be noticed that the spin contributions in Eq. (83) appear both explicitly, via the terms involving $s_{ij}$, and implicitly, through, e.g., the specific internal energy $\varPi$ (see Eq. (55)).

For our forthcoming analysis, it is useful to define a new set of STF mass-type radiative moments $I_L^{\mathrm{rad}}$ and current-type radiative moments $J_L^{\mathrm{rad}}$ according to

87 $$\begin{aligned} U_L&\equiv \overset{(l)}{I}{}_{L}^{\mathrm{rad}},\qquad V_L \equiv \overset{(l)}{J}{}_{L}^{\mathrm{rad}}. \end{aligned}$$

Bearing in mind the above equations jointly with Eqs. (13), (14), and (83), the 1PN fine-grained form of $I_{ij}^{\mathrm{rad}}$, and the leading-order expressions of $I_{ijk}^{\mathrm{rad}}$, $I_{ijkl}^{\mathrm{rad}}$, $J_{ij}^{\mathrm{rad}}$, and $J_{ijk}^{\mathrm{rad}}$ are given by, respectively,

88 $$\begin{aligned} I_{ij}^{\mathrm{rad}}(t)&= \int \mathrm{d}^3\varvec{x} \Biggl [ x^{\langle ij\rangle } \left( \rho ^{\star \star } + \rho _\mathrm{v}\right) \nonumber \\&\quad + \dfrac{4}{c^2} \left( s_{il}x^j+s_{jl}x^i-\dfrac{2}{3}\delta ^{ij}s_{kl}x^k \right) v^l \Biggr ] \nonumber \\&\quad + \dfrac{1}{14c^2} \dfrac{\mathrm{d}^2}{\mathrm{d}t^2} \int \mathrm{d}^3\varvec{x} \, x^{\langle ij\rangle } \varvec{x}^2 \rho ^\star \nonumber \\&\quad - \dfrac{20}{21c^2} \dfrac{\mathrm{d}}{\mathrm{d}t} \int \mathrm{d}^3\varvec{x} \Biggl [ x^{\langle ijk\rangle } v^k \rho ^\star \nonumber \\&\quad +\dfrac{7}{5} \left( s_{il}x^j+s_{jl}x^i\right) x^l \Biggr ] +\mathrm{O}(c^{-3}), \end{aligned}$$

89 $$\begin{aligned} I_{ijk}^{\mathrm{rad}}(t)&= \int \mathrm{d}^3\varvec{x}\, x^{\langle ijk\rangle }\rho ^\star +\mathrm{O}(c^{-2}), \end{aligned}$$

90 $$\begin{aligned} I_{ijkl}^{\mathrm{rad}}(t)&= \int \mathrm{d}^3\varvec{x}\, x^{\langle ijkl\rangle }\rho ^\star +\mathrm{O}(c^{-2}), \end{aligned}$$

91 $$\begin{aligned} J_{ij}^{\mathrm{rad}}(t)&= \int \mathrm{d}^3\varvec{x}\, \epsilon ^{kl\langle i} x^{j\rangle k} \rho ^\star v^l \nonumber \\&\quad \times \dfrac{1}{2} \int \mathrm{d}^3\varvec{x} \left[ \left( 2\epsilon ^{kl(i}s_{j)l}\right) x^k + \left( 2\epsilon ^{kl(i} x^{j)}\right) s_{kl}\right] \nonumber \\&\quad +\mathrm{O}(c^{-2}), \end{aligned}$$

92 $$\begin{aligned} J_{ijk}^{\mathrm{rad}}(t)&= \int \mathrm{d}^3\varvec{x}\Bigl [\rho ^\star x^{\langle i} x^j \epsilon ^{k \rangle lp} x^l v^p+ 2 s_{np} \delta _n^{\langle i} x^j \epsilon ^{k \rangle lp} x^l \nonumber \\&\quad + s_{lp} x^{\langle i} x^j \epsilon ^{k \rangle lp} \Bigr ] +\mathrm{O}(c^{-2}), \end{aligned}$$

where we have employed the Gauss theorem to discard the integrals containing a total divergence (recall that, in our model, both the spin angular momentum tensor (35) and the metric energy-momentum tensor (42) have compact support) and, in Eq. (91), the identity93

#### The ADM mass and the center of mass

In Ref. [66], we have demonstrated that the mass monopole moment *I*, which can be read off Eq. (14a) with $l=0$, gives rise to a generalized notion of * ADM mass* (or *total mass-energy*) of the fluid system and can be written as

94 $$\begin{aligned} I\left( t\right)&\equiv M_\mathrm{ADM}\left( t\right) = \int \mathrm{d}^3 \varvec{x} \left[ \sigma + \dfrac{1}{c^2}\left( \dfrac{1}{2} \sigma U -\sigma _{ii}\right) \right] \nonumber \\&\quad + \mathrm{O}(c^{-4}), \end{aligned}$$

and, in addition, satisfies

95 $$\begin{aligned} \dfrac{\mathrm{d}}{\mathrm{d}t} M_\mathrm{ADM}=0. \end{aligned}$$

In the case of the Weyssenhoff fluid, we find, with the help of Eq. (83),

96 $$\begin{aligned} M_\mathrm{ADM} = \int \mathrm{d}^3 \varvec{x} \, \rho ^{\star \star } + \mathrm{O}(c^{-4}), \end{aligned}$$

where we have exploited Gauss theorem to discard total derivatives occurring in $\sigma$ and $\sigma _{ii}$ (cf. Eqs. (83a) and (83c)). For this reason, Eq. (96) resembles formally the GR expression [17]. The ADM mass (96) assumes the equivalent form

97 $$\begin{aligned} M_\mathrm{ADM} \equiv m^\mathrm{mat} + \dfrac{\mathscr {E}}{c^2}, \end{aligned}$$

where

98 $$\begin{aligned} \mathscr {E} = \mathscr {T} + \mathscr {E}_\mathrm{p} + \mathscr {E}_\mathrm{int} + \mathrm{O}(c^{-2}), \end{aligned}$$

and

99a $$\begin{aligned} m^\mathrm{mat}&\equiv \int \mathrm{d}^3 \varvec{x} \, \rho ^{\star }, \end{aligned}$$

99b $$\begin{aligned} \mathscr {T}&\equiv \dfrac{1}{2} \int \mathrm{d}^3 \varvec{x} \, \rho ^{\star } v^2, \end{aligned}$$

99c $$\begin{aligned} \mathscr {E}_\mathrm{p}&\equiv -\dfrac{1}{2} \int \mathrm{d}^3 \varvec{x} \, \rho ^{\star } U, \end{aligned}$$

99d $$\begin{aligned} \mathscr {E}_\mathrm{int}&\equiv \int \mathrm{d}^3 \varvec{x} \, \rho ^{\star } \varPi , \end{aligned}$$

denote the total material mass, the translational kinetic energy, the total gravitational potential energy, and the total internal energy of the fluid system, respectively. It should be noted that the contribution of the spin kinetic energy is included in Eq. (99d). Furthermore, Eqs. (71), (76b), and

100 $$\begin{aligned} \dfrac{\mathrm{d}\varPi }{\mathrm{d}t}= \dfrac{P}{\rho ^{\star 2}}\dfrac{\mathrm{d}\rho ^\star }{\mathrm{d}t}+ \mathrm{O}(c^{-2}), \end{aligned}$$

which can be obtained through Eqs. (57) and (72), assure the constancy of the ADM mass (97), in agreement with Eq. (95).

The case $l=1$ of Eq. (14a) yields the definition of the *dipole moment*, which for the Weyssenhoff fluid becomes

101 $$\begin{aligned} I_i(t)= \int \mathrm{d}^3 \varvec{x} \,x^i \rho ^{\star \star } + \dfrac{2}{c^2} \int \mathrm{d}^3 \varvec{x}\, s_{il}v^l + \mathrm{O}(c^{-4}), \end{aligned}$$

where we have exploited the Gauss theorem.

The *position of the center of mass* of the fluid system, which is defined as $\mathscr {R}^i \equiv I_i/M_\mathrm{ADM}$ [17, 80], reads as

102 $$\begin{aligned} \mathscr {R}^i&= \dfrac{1}{M_\mathrm{ADM}}\left[ \int \mathrm{d}^3 \varvec{x} \,x^i \rho ^{\star \star } + \dfrac{2}{c^2} \int \mathrm{d}^3 \varvec{x}\, s_{il}v^l \right] + \mathrm{O}(c^{-4}),\nonumber \\ \end{aligned}$$

and satisfies the relation

103 $$\begin{aligned} M_\mathrm{ADM} \dfrac{\mathrm{d}\mathscr {R}^i}{\mathrm{d}t}= \mathscr {P}^i + \mathrm{O}(c^{-4}), \end{aligned}$$

$\mathscr {P}^i$ being the *conserved ADM three-momentum* of the system (see Ref. [66] for further details).

It should be noted that an explicit spin correction term occurs in Eq. (102). This is similar to the contribution appearing in the analysis of spinning bodies within GR [17, 103]. However, we recall the basic difference between EC model and Einstein theory, as in the former the spin refers to the intrinsic *quantum* angular momentum of elementary particles, while in the latter the spin is related to a macroscopic rotation [78].

#### The coarse-grained description

We are now ready to apply the results of the previous sections to the framework where the fine-grained description of the Weyssenhoff fluid is replaced by the coarse-grained picture involving separated fluid elements, which are characterized by a small number of variables [17, 102]. Therefore, we consider a setup where the fluid distribution breaks up into a collection of *N* weakly self-gravitating, slowly moving, widely separated, and isolated spinning components surrounded by vacuum regions of space. Each component, referred to as body, is assigned a label $A=1,2,\dots ,N$, and, in our hypotheses, the ratio $\alpha \equiv \lambda /\delta \ll 1$ ($\lambda$ and $\delta$ being the typical size of *A* and the typical separation between the bodies, respectively). The coordinate fluid density can be expressed as $\rho ^\star = \sum _{A} \rho ^\star _A$, where the sum extends over each body, and $\rho ^\star _A$ vanishes everywhere except within the volume occupied by the body *A*. Furthermore, the (conserved) material mass of *A* is $m^\mathrm{mat}_A = \int _A \mathrm{d}^3 \varvec{x} \, \rho ^\star$, the domain of integration being a time-independent portion of the three-dimensional space which extends slightly beyond the volume occupied by the body *A* and does not include nor intersect another body within the system (recall that $\rho ^\star =\rho ^\star _A$ within such a domain).

Similarly, the spin density tensor $s_{ij}$ is zero in the vacuum exterior of the bodies, whereas $s_{ij}=s^A_{ij}$ inside the volume of the body *A*. The spin vector $\varvec{s}_A$ of *A* is defined by

104 $$\begin{aligned} \epsilon _{jki}s_A^i(t)=\int _A \mathrm{d}^3 \varvec{x} \, s^A_{jk}, \end{aligned}$$

and, owing to Eq. (76c), is conserved modulo $\mathrm{O}\left( c^{-2}\right)$ corrections, i.e.,

105 $$\begin{aligned} \dfrac{\mathrm{d}}{\mathrm{d}t}s^i_A = \mathrm{O}\left( c^{-2}\right) . \end{aligned}$$

Driven by Eqs. (102) and (96), the center of mass worldline $r^i_A\left( t\right)$ of the body *A* is defined as

106 $$\begin{aligned} r^i_A\left( t\right)&= \dfrac{1}{m_A} \int _A \mathrm{d}^3 \varvec{x} \,x^i \rho _A^\star \left[ 1+\dfrac{1}{c^2} \left( \dfrac{w_A^2}{2} + \varPi _A -\dfrac{u_A}{2}\right) \right] \nonumber \\&\quad + \dfrac{2}{m_A c^2} \int _A \mathrm{d}^3 \varvec{x} \, s^A_{il}w^l_A +\mathrm{O}(c^{-4}), \end{aligned}$$

where

107 $$\begin{aligned} m_A&=\int _A \mathrm{d}^3 \varvec{x} \, \rho _A^\star \left[ 1+\dfrac{1}{c^2} \left( \dfrac{w_A^2}{2} + \varPi _A -\dfrac{u_A}{2}\right) \right] \nonumber \\&\quad + \mathrm{O}(c^{-4}), \end{aligned}$$

is the total mass-energy of the body, which, along the same lines as for $M_\mathrm{ADM}$, can be shown to be conserved. In Eqs. (106) and (107), $\varPi _A$ denotes the specific internal energy of *A*; furthermore, $u_A$ is the internal (self-gravity) potential, which is readily obtained from the Newtonian potential (86); finally, upon introducing the vector $y^i_A \equiv x^i -r^i_A\left( t\right)$, measuring the position of a fluid element relative to the center of mass $r^i_A\left( t\right)$, $w^i_A$ is given by $w^i_A \equiv \tfrac{\mathrm{d}}{\mathrm{d}t} y^i_A = v^i - v^i_A\left( t\right)$, and represents the velocity of this fluid element relative to the body velocity $v^i_A\left( t\right) \equiv \tfrac{\mathrm{d}}{\mathrm{d}t} r^i_A \left( t\right)$. The above definitions permit renormalizing the internal selfgravity of *A* into the mass $m_A$.

#### The point-particle limit of the radiative moment

At this stage, we have all the ingredients needed to perform the point-particle limit of the radiative moments (88)–(92). For this reason we employ the same techniques as in Ref. [102].

In EC theory, the following crucial identities hold:

108a $$\begin{aligned}&\int _A \mathrm{d}^3 \varvec{y}_A y^i_A \rho ^\star _A \left[ 1+\dfrac{1}{c^2}\left( \dfrac{w_A^2}{2}+\varPi _A -\dfrac{u_A}{2}\right) \right] \nonumber \\&\quad + \dfrac{2}{c^2} \int _A \mathrm{d}^3 \varvec{y}_A s^A_{il}w^l_A = \mathrm{O}\left( c^{-4}\right) , \end{aligned}$$

108b $$\begin{aligned}&\int _A \mathrm{d}^3 \varvec{y}_A y^i_A \rho ^\star _A = \mathrm{O}\left( c^{-2}\right) , \end{aligned}$$

108c $$\begin{aligned}&\int _A \mathrm{d}^3 \varvec{y}_A w^i_A \rho ^\star _A = \mathrm{O}\left( c^{-2}\right) , \end{aligned}$$

108d $$\begin{aligned}&\dfrac{1}{2} \dfrac{\mathrm{d}^2}{\mathrm{d}t^2} \int _A \mathrm{d}^3 \varvec{y}_A \varvec{y}^2_A \rho ^\star _A \nonumber \\&\quad = \int _A \mathrm{d}^3 \varvec{y}_A \rho ^\star _A \left( w_A^2 -\dfrac{u_A}{2}+\dfrac{3P_A}{\rho ^\star _A}\right) + \mathrm{O}\left( c^{-2}\right) , \end{aligned}$$

where $P_A$ is the pressure within body *A* and all functions are supposed to depend on *t* and the position $\varvec{y}_A + \varvec{r}_A(t)$. Equation (108a) is a consequence of Eqs. (106) and (107); Eq. (108b) stems from Eq. (108a) and, in turn, Eq. (108c) can be obtained by evaluating the time derivative of Eq. (108b); finally, Eq. (108d) derives from the virial theorem, which reads as (cf. Eq. (83))

109 $$\begin{aligned} \dfrac{1}{2} \dfrac{\mathrm{d}^2}{\mathrm{d}t^2} \int \mathrm{d}^3 \varvec{x}\, \varvec{x}^2 \sigma = \int \mathrm{d}^3 \varvec{x} \left( \sigma _{jj} -\dfrac{1}{2}\sigma U \right) + \mathrm{O}\left( c^{-2}\right) . \end{aligned}$$

By supposing that all bodies are *spherically symmetric* and in *static equilibrium*, we find that the point-particle counterpart of the radiative mass quadrupole moment (88) reads as110moreover, for the the mass octupole and current quadrupole, the point-particle procedure yields, respectively,

111 $$\begin{aligned}&I^\mathrm{rad}_{ijk}=\sum _A m_A r_A^{\langle i}r_A^jr_A^{k\rangle }+\mathrm{O}\left( c^{-2}\right) , \end{aligned}$$

112 $$\begin{aligned}&J^\mathrm{rad}_{ij} =\sum _A m_A \epsilon ^{kl\langle i}r_A^{j\rangle }r_A^kv_A^l \nonumber \\&+\dfrac{1}{2}\sum _A \left[ 3 \left( s^i_A r^j_A+s^j_A r^i_A\right) -2 \delta ^{ij} \varvec{s}_A \cdot \varvec{r}_A\right] +\mathrm{O}\left( c^{-2}\right) ; \end{aligned}$$

the mass $2^4$-pole and the current octupole give, respectively,

113 $$\begin{aligned} I^\mathrm{rad}_{ijkl}&=\sum _A m_A r_A^{\langle i}r_A^jr^k_Ar_A^{l\rangle }+\mathrm{O}\left( c^{-2}\right) , \end{aligned}$$

114 $$\begin{aligned} J^\mathrm{rad}_{ijk}&=\sum _A \Bigl [ m_A r_A^{\langle i} r^j_A \epsilon ^{k \rangle lp} r_A^l v^p_A+ 2 \Bigl (r^n_A s^q_A \, \delta _n^{\langle i}r^j_A \delta ^{k \rangle }_q \nonumber \\&\qquad -\varvec{r}_A \cdot \varvec{s}_A \, \delta _n^{\langle i}r^j_A \delta ^{k \rangle }_n + s_A^q \, r_A^{\langle i} r^j_A \delta _q^{k \rangle }\Bigr ) \Bigr ] +\mathrm{O}\left( c^{-2}\right) . \end{aligned}$$

Equations (110)–(112) are required for the computation of the instantaneous luminosity (18), whereas the whole set of radiative moments (110)–(114) appears in the asymptotic waveform (16) (cf. Eq. (87)). It is clear that EC theory brings in explicit corrections due to the spin $\varvec{s}_A$ of the body *A* in $I^\mathrm{rad}_{ij}$, $J^\mathrm{rad}_{ij}$, and $J^\mathrm{rad}_{ijk}$.

We recall that, in deriving Eqs. (110)–(114), we have neglected terms $\mathrm{O}\left( \alpha ^{2}\right)$.

### Binary systems

In this section, we set out the features of the special case of binary systems, i.e., a collection of $N=2$ weakly self-gravitating, slowly moving, and widely separated spinning fluid bodies having masses $m_1$, $m_2$ (with $m_1\ge m_2$), position vectors $\varvec{r}_1$, $\varvec{r}_2$, velocities $\varvec{v}_1$, $\varvec{v}_2$, and spin vectors $\varvec{s}_1$, $\varvec{s}_2$. Let $M\equiv m_1+m_2$, $\mu \equiv \tfrac{m_1m_2}{M}$, and $\nu \equiv \tfrac{\mu }{M}$ denote the total mass, the reduced mass, and the symmetric mass ratio of the system, respectively. As in the Newtonian framework [17, 104], the study of the dynamics is simplified once the origin of the coordinate frame is attached to the barycenter $\mathscr {R}^i$ of the system and the position of each body is determined in terms of their separation vector. Accordingly, we introduce the instantaneous relative position vector $\varvec{R}$ and the instantaneous relative velocity vector $\varvec{V}$^4^ of the two objects

115a $$\begin{aligned} \varvec{R}(t)&\equiv \varvec{r}_1(t)-\varvec{r}_2(t), \end{aligned}$$

115b $$\begin{aligned} \varvec{V}(t)&\equiv \dfrac{\mathrm{d}}{\mathrm{d}t}\varvec{R}(t)=\varvec{v}_1(t)-\varvec{v}_2(t). \end{aligned}$$

Moreover, starting from Eq. (102), we find that, within the coarse-grained description of the fluid and resorting to the same techniques as in the last sections, the position of the barycenter of a binary system is defined by (we discard, like before, $\mathrm{O}\left( \alpha ^{2}\right)$ corrections)

116 $$\begin{aligned} M_\mathrm{ADM}\mathscr {R}^i&= m_1 \Biggl [ 1+ \dfrac{1}{2c^2} \Biggl ( v_1^2 - \dfrac{Gm_2}{R}\Biggl )\Biggr ]r^i_1 \nonumber \\&\quad + \dfrac{2}{c^2}\left( \varvec{v}_1 \times \varvec{s}_1\right) ^i \nonumber \\&\quad + m_2 \Biggl [ 1 + \dfrac{1}{2c^2} \Biggl ( v_2^2 - \dfrac{Gm_1}{R}\Biggl )\Biggr ]r^i_2 \nonumber \\&\quad + \dfrac{2}{c^2}\left( \varvec{v}_2 \times \varvec{s}_2\right) ^i +\mathrm{O}\left( c^{-4}\right) . \end{aligned}$$

By employing a post-Galilean transformation (i.e., a particular subclass of general PN transformations [17]), it is always possible to define the center of mass frame of the system by setting $\mathscr {R}^i=0$ and $\mathscr {P}^i=0$. Therefore, by means of Eq. (116), we find that in a mass-centered coordinate system the motion of the bodies is related to their relative motion by the following relations:

117a $$\begin{aligned} \varvec{r}_1(t)&=\left[ \frac{\mu }{m_1}+\frac{\mu (m_1-m_2)}{2M^2c^2}\left( V^2-\frac{GM}{R}\right) \right] \varvec{R}(t) \nonumber \\&\quad +\frac{2 \nu }{c^2}\left[ \dfrac{\varvec{s}_1(t)}{m_1} -\dfrac{\varvec{s}_2(t)}{m_2}\right] \times \varvec{V}(t)+\mathrm{O}\left( c^{-4}\right) , \end{aligned}$$

117b $$\begin{aligned} \varvec{r}_2(t)&=\left[ -\frac{\mu }{m_2}+\frac{\mu (m_1-m_2)}{2M^2c^2}\left( V^2-\frac{GM}{R}\right) \right] \varvec{R}(t)\nonumber \\&\quad +\frac{2 \nu }{c^2}\left[ \dfrac{\varvec{s}_1(t)}{m_1} -\dfrac{\varvec{s}_2(t)}{m_2}\right] \times \varvec{V}(t)+\mathrm{O}\left( c^{-4}\right) . \end{aligned}$$

If we replace Eq. (117) in Eqs. (110)–(114), we find that the general form of the radiative moments for binaries of spinning objects reads as

118 $$\begin{aligned} I^\mathrm{rad}_{ij}&=\mu R_{\langle ij\rangle }\left[ 1+\frac{3}{2c^2}(1-3\nu )V^2-\frac{(1-2\nu )}{c^2}\frac{GM}{R}\right] \nonumber \\&\quad -\frac{\mu (1-3\nu )}{21c^2}\left[ 20\frac{\mathrm{d}}{\mathrm{d}t}(V_kR_{\langle ijk\rangle })-\frac{3}{2}\frac{\mathrm{d}^2}{\mathrm{d}t^2}(R^2R_{\langle ij\rangle })\right] \nonumber \\&\quad +\frac{8\mu ^2}{c^2}\left\{ \frac{(\varvec{V}\times \varvec{s}_1)^{\langle i}R^{j\rangle }}{m_1^2}+\frac{(\varvec{V}\times \varvec{s}_2)^{\langle i}R^{j\rangle }}{m_2^2}\right. \nonumber \\&\quad \left. -\frac{1}{3}\frac{\mathrm{d}}{\mathrm{d}t}\left[ \frac{(\varvec{R}\times \varvec{s}_1)^{(i}R^{j)}}{m_1^2}+\frac{(\varvec{R}\times \varvec{s}_2)^{(i}R^{j)}}{m_2^2}\right] \right\} \nonumber \\&\quad +\mathrm{O}\left( c^{-3}\right) , \end{aligned}$$

119 $$\begin{aligned} I^\mathrm{rad}_{ijk}&=-\mu \sqrt{1-4\nu }R_{\langle ijk\rangle }+\mathrm{O}\left( c^{-2}\right) ,\end{aligned}$$

120 $$\begin{aligned} J^\mathrm{rad}_{ij}&=-\mu \sqrt{1-4\nu }\epsilon _{kl\langle i}R_{j\rangle k}V_l\nonumber \\&\qquad +3\mu \left( \frac{s_1^{\langle i}R^{j\rangle }}{m_1}-\frac{s_2^{\langle i}R^{j\rangle }}{m_2}\right) +\mathrm{O}\left( c^{-2}\right) , \end{aligned}$$

121 $$\begin{aligned} I^\mathrm{rad}_{ijkl}&=\mu (1-3\nu )R_{\langle ijkl\rangle } +\mathrm{O}\left( c^{-2}\right) , \end{aligned}$$

122 $$\begin{aligned} J^\mathrm{rad}_{ijk}&=\mu (1-3\nu )R_{\langle ij}\epsilon _{k\rangle lp}R_l V_p\nonumber \\&\quad +\sum _{A=1}^{2}\frac{2\mu ^2}{m_A^2} \Bigl (R^n s^q_A \, \delta _n^{\langle i}R^j \delta ^{k \rangle }_q-\varvec{R} \cdot \varvec{s}_A \, \delta _n^{\langle i}R^j \delta ^{k \rangle }_n \nonumber \\&\quad + s_A^q \, R^{\langle i} R^j \delta _q^{k \rangle }\Bigr )+\mathrm{O}\left( c^{-2}\right) . \end{aligned}$$

The above equations completely determine the 1PN generation of GWs from binary systems in EC theory.

## First application to binary neutron star systems

The theoretical pattern developed in the previous sections will be now applied to the study of binary NSs. A full analysis requires the knowledge of the 1PN dynamics in EC theory, which, at the moment, is not at our disposal. Despite that, we can provide a first estimation by following a *hybrid approach*. We exploit the EC definition of center of mass and the general expression of the EC radiative moments obtained in Sect. 4.2, along with the conservation equation of the spin vector (cf. Eqs. (76c) and (105)); furthermore, we consider the *quasi-elliptic 1PN-accurate GR motion of a binary system* determined by Damour and Deruelle [104] (see Sect. 5.1).

We note that, in the full EC framework, only the time derivatives of the radiative mass quadrupole moment $I_{ij}^\mathrm{rad}$ will contain new $\mathrm{O}(c^{-2})$ spin-dependent terms, whereas the other moments will remain unaffected. Indeed, as pointed out before, the EC 0PN-accurate translational motion coincides with the Newtonian Euler equation owing to the Frenkel condition (cf. Eq. (76b)).

In this hybrid setup, we obtain the explicit expressions of the flux and the gravitational waveform (see Sect. 5.2). We conclude the section with a numerical estimate concerning the EC corrections by examining binary NS systems (see Sect. 5.3).

Any effect of GW back-reaction on the source dynamics will be neglected. This hypothesis, which is valid to a good approximation in some astrophysical GW sources (see e.g. Refs. [105, 106]), permits to derive a first model for the description of GW phenomena in EC theory. Hereafter, we no longer mention $\mathrm{O}\left( c^{-n}\right)$ terms.

### The Damour–Deruelle solution

We consider, within the hybrid approach set forth above, a binary system consisting of two PN widely separated spinning bodies having masses $m_1$, $m_2$ and spin vectors $\varvec{s}_1$, $\varvec{s}_2$. As pointed out before, we assume that their dynamics is governed by the Damour–Deruelle solution, which we now briefly outline.

The problem of solving the motion of the binary system can be reduced to the simpler equivalent task of determining the relative motion in the PN center of mass frame (which can be defined by means of the results of Sect. 4.2). For this reason, given a harmonic coordinate system, we define, in the same way as before, the instantaneous relative position vector $\varvec{R}$ and the instantaneous relative velocity vector $\varvec{V}$ (cf. Eq. (115)).

The total energy *E* and the total angular momentum $\varvec{J}$ of the system (which are conserved in the Damour–Deruelle dynamics) read as, respectively,

123 $$\begin{aligned} E&=\frac{1}{2}V^2-\frac{GM}{R}+ \dfrac{1}{c^2} \Biggl \{ \frac{3}{8}V^4 (1-3\nu ) \nonumber \\&\quad +\frac{GM}{2R}\left[ (3+\nu )V^2+\nu \left( \frac{\varvec{R}\cdot \varvec{V}}{R}\right) ^2+\frac{GM}{R}\right] \Biggr \}, \end{aligned}$$

124 $$\begin{aligned} \varvec{J}&=\varvec{R}\times \varvec{V}\left\{ 1+\dfrac{1}{c^2}\left[ \frac{(1-3\nu )}{2}V^2+(3+\nu )\frac{GM}{R}\right] \right\} . \end{aligned}$$

The motion takes place in the plane orthogonal to $\varvec{J}$, which is henceforth supposed to be directed along the *z*-axis. We can thus introduce polar coordinates $(R,\varphi )$ and write the equation of the PN *relative orbit* as

125 $$\begin{aligned} R(\varphi )=\left( a_\mathrm{R}-\frac{G\mu }{2c^2}\right) \frac{1-e_\varphi ^2}{1+e_\varphi \cos \left( \frac{\varphi -\varphi _\mathrm{in}}{K}\right) }+\frac{G\mu }{2c^2}, \end{aligned}$$

where $\varphi _\mathrm{in}$ is the initial angle and the following orbital parameters have been introduced:

126a $$\begin{aligned} a_R&=-\frac{GM}{2E}\left[ 1-\frac{1}{2}(\nu -7)\frac{E}{c^2}\right] , \end{aligned}$$

126b $$\begin{aligned} e_R&=\left\{ 1+\frac{2E}{G^2M^2}\frac{\left[ 1+\frac{5}{2}\left( \nu -3\right) \frac{E}{c^2}\right] }{\left[ J^2+(\nu -6)\frac{G^2M^2}{c^2}\right] ^{-1}}\right\} ^{1/2}, \end{aligned}$$

126c $$\begin{aligned} e_\varphi&=e_R\left( 1+\frac{G\mu }{2a_Rc^2}\right) , \end{aligned}$$

126d $$\begin{aligned} K&=\frac{J}{(J^2-6G^2M^2/c^2)^{1/2}}. \end{aligned}$$

### The flux and the gravitational waveform

It follows from the definition (87) that the instantaneous luminosity (18) attains the equivalent form (restoring, for a while, the $\mathrm{O}(c^{-n})$ terms)

127 $$\begin{aligned} \mathcal {F}(t)&= \dfrac{G}{c^5} \Biggl \{\dfrac{1}{5}\overset{(3)}{I}{}_{ij}^{\mathrm{rad}}\overset{(3)}{I}{}^{\mathrm{rad}}_{ij} + \dfrac{1}{c^2} \Biggl [ \dfrac{1}{189} \overset{(4)}{I}{}^{\mathrm{rad}}_{ijk} \overset{(4)}{I}{}^{\mathrm{rad}}_{ijk} \nonumber \\&\quad + \dfrac{16}{45} \overset{(3)}{J}{}^\mathrm{rad}_{ij}\overset{(3)}{J}{}_{ij}^\mathrm{rad} \Biggr ] + \mathrm{O}(c^{-4}) \Biggr \}, \end{aligned}$$

whereas the asymptotic amplitude (16) becomes

128 $$\begin{aligned} \mathscr {H}_{ij}^\mathrm{TT}(x^\mu )&= \dfrac{2G}{c^4 \vert \varvec{x}\vert } \mathscr {P}_{ijkl}(\varvec{n}) \Biggr \{ \overset{(2)}{I}{}_{kl}^{\mathrm{rad}}(u) \nonumber \\&\quad + \dfrac{1}{c} \left[ \dfrac{1}{3} n_a \overset{(3)}{I}{}_{kla}^{\mathrm{rad}}(u) +\dfrac{4}{3} n_b \epsilon _{ab(k} \overset{(2)}{J}{}_{l)a}^{\mathrm{rad}}(u) \right] \nonumber \\&\quad +\dfrac{1}{c^2} \left[ \dfrac{1}{12}n_{a}n_{b} \overset{(4)}{I}{}_{klab}^{\mathrm{rad}}(u) \right. \nonumber \\&\quad \left. + \dfrac{1}{2}n_{b}n_{c} \epsilon _{ab(k} \overset{(3)}{J}{}_{l)ac}^{\mathrm{rad}}(u) \right] + \mathrm{O}(c^{-3}) \Biggr \}, \end{aligned}$$

where $u\equiv t-\vert \varvec{x}\vert /c$, $\varvec{n}\equiv \varvec{x}/\vert \varvec{x}\vert$, and the radiative moments have been derived in Eqs. (118)–(122). We also note that in Eqs. (127) and (128) we have exploited the fact that, at this order, there is no difference between the harmonic and the radiative coordinates [80, 81].

At this stage, we can compute $\mathcal {F}$ and $\mathscr {H}_{11}^\mathrm{TT}$ by exploiting the Damour–Deruelle solution.

The *instantaneous luminosity* (127) can be written as

129 $$\begin{aligned} \mathcal {F}(t)=\mathcal {F}_\mathrm{GR}(t)+\mathcal {F}_\mathrm{EC}(t), \end{aligned}$$

where the expression of the GR flux can be found in Ref. [102], while the EC contribution can be obtained after a lengthy calculation by exploiting the results of Sects. 4.2 and 5.1 . In the hypotheses that the motion occurs in the *xy*-plane (i.e., $R_z=V_z=0$) and the spins of the two bodies are aligned with the total angular momentum $\varvec{J}$ (i.e., $s_{x1}=s_{x2}=s_{y1}=s_{y2}=0$), we find for the EC luminosity

130 $$\begin{aligned} \mathcal {F}_\mathrm{EC}(t)&= \frac{8 G^3}{15 c^7 R^8} \biggr \{8 G \mu M R \left( m_1^2 s_\mathrm{z2}+m_2^2 s_\mathrm{z1}\right) \nonumber \\&\quad \times (R_x V_y-R_y V_x)+\frac{m_1^3 s_\mathrm{z2}}{M} \biggr [m_2 R_x^3 \left( 4 V_x^2 V_y\right. \nonumber \\&\quad \left. +58 V_y^3\right) +R_x^2 \left( 3 s_\mathrm{z2} \left( 4 V_x^2+V_y^2\right) \right. \nonumber \\&\quad \left. -2 m_2 R_y V_x \left( 2 V_x^2+83 V_y^2\right) \right) \nonumber \\&\quad +2 R_x R_y V_y \left( m_2 R_y \left( 83 V_x^2+2 V_y^2\right) +9 s_\mathrm{z2} V_x\right) \nonumber \\&\quad +R_y^2 \left( 3 s_\mathrm{z2} \left( V_x^2+4 V_y^2\right) -2 m_2 R_y V_x \left( 29 V_x^2\right. \right. \nonumber \\&\quad \left. \left. +2 V_y^2\right) \right) \biggr ]+\frac{3 m_2^3 s_\mathrm{z1}^2 }{M}\biggr [R_x^2 \left( 4 V_x^2+V_y^2\right) \nonumber \\&\quad +6 R_x R_y V_x V_y+R_y^2 \left( V_x^2+4 V_y^2\right) \biggr ]\nonumber \\&\quad +\mu m_1 \biggr [R_x^2 \left( 4 V_x^2+V_y^2\right) +6 R_x R_y V_x V_y\nonumber \\&\quad +R_y^2 \left( V_x^2+4 V_y^2\right) \biggr ] \left( -2 m_2 R_x V_y (s_\mathrm{z1}+s_\mathrm{z2})\right. \nonumber \\&\quad \left. +2 m_2 R_y s_\mathrm{z1} V_x+2 m_2 R_y s_\mathrm{z2} V_x-6 s_\mathrm{z1} s_\mathrm{z2}+3 s_\mathrm{z2}^2\right) \nonumber \\&\quad +\mu m_2 s_\mathrm{z1} \biggr [V_x^2 \left( 3 \left( 4 R_x^2+R_y^2\right) (s_\mathrm{z1}-2 s_\mathrm{z2})\right. \nonumber \\&\quad \left. -2 m_2 R_y V_x \left( 2 R_x^2+29 R_y^2\right) \right) \nonumber \\ {}&\quad +V_y^2 \left( 3 \left( R_x^2+4 R_y^2\right) \right. \nonumber \\&\quad \left. \times (s_\mathrm{z1}-2 s_\mathrm{z2})-2 m_2 R_y V_x \left( 83 R_x^2+2 R_y^2\right) \right) \nonumber \\&\quad +2 R_x V_x V_y \left( 2 m_2 R_x^2 V_x+83 m_2 R_y^2 V_x\right. \nonumber \\&\quad \left. +9 R_y (s_\mathrm{z1}-2 s_\mathrm{z2})\right) \nonumber \\&\quad +2 m_2 R_x V_y^3 \left( 29 R_x^2+2 R_y^2\right) \biggr ]\biggr \}. \end{aligned}$$

The general form of the gravitational waveform can be obtained after a long calculation by considering the time derivatives of the radiative moments appearing in Eq. (128). In this section, we give the component $\mathscr {H}_{11}^\mathrm{TT}$ of the asymptotic amplitude in the hypotheses that the GW propagates along the direction $\varvec{n}=(0,0,1)$ and, like before, the spins of the two bodies are aligned and orthogonal to the *xy*-plane of motion. The resulting expression can be written as the sum of the GR and the EC contributions, i.e., (in order to ease the notation, henceforth we write $\mathscr {H}_{11} \equiv \mathscr {H}_{11}^\mathrm{TT}$)

131 $$\begin{aligned} \mathscr {H}_{11}(t)&=\mathscr {H}_{11}^\mathrm{GR}(t)+\mathscr {H}_{11}^\mathrm{EC}(t), \end{aligned}$$

where

132 $$\begin{aligned} \mathscr {H}_{11}^\mathrm{GR}(t)&= \frac{G \mu }{3d_\mathrm{so}R^6c^6} \Biggl \{29G^2 M^2 \left( R_x^4-R_y^4\right) +3R^6 \nonumber \\&\quad \times \left( V_x^2-V_y^2\right) \left[ 2c^2 + V^2 \left( \dfrac{\mu ^2}{m_1^2}+\dfrac{\mu ^2}{m_2^2}-\nu \right) \right] \nonumber \\&\quad -3G M R \Biggl [2c^2 \left( R_x^4 -R_y^4\right) +\left( \dfrac{\mu ^2}{m_2^2} + \dfrac{\mu ^2}{m_1^2}\right) \nonumber \\&\quad \times \Bigl (2R_x R_y V_x V_y \left( R_y^2-R_x^2\right) \nonumber \\ {}&\quad +3R_x^2 R_y^2 \left( V_y^2-V_x^2\right) + R_x^4 \left( 2V_y^2-3V_x^2\right) \nonumber \\&\quad +R_y^4 \left( 3V_y^2-2V_x^2\right) \Bigr ) +\nu \Bigl ( 2R_x R_y V_x V_y \nonumber \\&\quad \times \left( R_x^2-R_y^2\right) -17R_x^2 R_y^2 \left( V_x^2-V_y^2\right) \nonumber \\&\quad + R_x^4 \left( 7V_y^2-8V_x^2\right) +R_y^4 \left( 8V_y^2-7V_x^2\right) \Bigr ) \Biggr ]\Biggr \}, \end{aligned}$$

133 $$\begin{aligned} \mathscr {H}_{11}^\mathrm{EC}(t)&=-\dfrac{8 \mu G^2}{c^6d_\mathrm{so}R^5} \left( \dfrac{m_2}{m_1}s_\mathrm{z1}+\dfrac{m_1}{m_2}s_\mathrm{z2}\right) \biggl [R_x^2 \left( 2R_xV_y \right. \nonumber \\&\qquad \left. -R_yV_x \right) +R_y^2 \left( 2R_yV_x-R_xV_y\right) \biggr ], \end{aligned}$$

$d_\mathrm{so}$ being the (constant) distance to the astrophysical source.

### Numerical estimates

In this section, we deal with binary NS systems and provide some numerical estimates of the contributions introduced by EC model in the flux and the waveform. In Sect. 5.3.1, we set the parameters which are necessary to perform the numerical computations. These are then discussed in Sect. 5.3.2. Hereafter, a dot signifies a differentiation with respect to the *t* variable.

#### Parameter setting

The set of *initial conditions* characterizing our numerical investigation is represented by $(R_\mathrm{in},\varphi _\mathrm{in},\dot{R}_\mathrm{in},\dot{\varphi }_\mathrm{in})$. The initial radius $R_\mathrm{in}$ is expressed in terms of $R_\mathrm{g}$, with $R_\mathrm{g}\equiv GM/c^2$; for the initial angle and radial velocity, we assume $\varphi _\mathrm{in}=0$ and $\dot{R}_\mathrm{in}=0$, respectively; $\dot{\varphi }_\mathrm{in}$ is a fraction of the Keplerian velocity, i.e.,

134 $$\begin{aligned} \dot{\varphi }_\mathrm{in}=\beta \sqrt{\frac{GM}{R_\mathrm{in}^3}}, \qquad 0<\beta \le 1, \end{aligned}$$

where for $\beta =1$ the Newtonian eccentricity $e_0$ vanishes (leading to circular orbits), whereas in the limiting case $\beta \rightarrow 0$ we have $e_0\rightarrow 1$. Therefore, given these premises, the initial conditions are specified once we assign *M*, $R_\mathrm{in}$, and $\beta$.

A crucial point of our analysis regards the spins of the NSs. These are modeled as follows

135 $$\begin{aligned} s_\mathrm{zi}= n\hbar \frac{4\pi }{3}\left( \frac{6Gm_i}{c^2}\right) ^3, \quad i=1,2, \end{aligned}$$

where, following Ref. [66], $n=10^{44}\ \text{ m}^{-3}$ is estimated as the inverse of the nucleon volume. Therefore, if the masses $m_1$ and $m_2$ are known, then the spin components $s_\mathrm{z1}$ and $s_\mathrm{z2}$ can be immediately calculated.

In order to gain useful information about the binary system’s dynamics, we determine the minimum, average, and maximum values of the relative radius (i.e., $R_\mathrm{min},R_\mathrm{av},R_\mathrm{max}$). Furthermore, to perform some consistency checks, we define a set of parameters, which must be less than 1 due to the hypotheses underlying our model; first of all, the slow-motion condition demands that we compute the maximum values $v_1^\mathrm{max}/c,v_2^\mathrm{max}/c$ attained by the ratios $v_1/c,v_2/c$, respectively (the velocities $v_1$ and $v_2$ of the two bodies can be obtained starting from Eq. (117)); to verify whether the two bodies remain widely separated, we calculate

136 $$\begin{aligned} \alpha _i=\frac{12Gm_i/c^2}{R_\mathrm{min}},\qquad i=1,2; \end{aligned}$$

finally, we monitor the strength of the gravitational field through the factor

137 $$\begin{aligned} \gamma =\frac{GM}{c^2R_\mathrm{min}}. \end{aligned}$$

The values of the aforementioned variables, along with other quantities characterizing the binary NS system to be investigated in Sect. 5.3.2, are listed in Table 1.

#### Discussion of the results

We consider a gravitational system consisting of two NSs, whose parameters can be found in Table 1.

**Table 1:** List of parameters of the binary NS system analyzed in Sect. 5.3.2

Parameters	Units	Values
$$m_1 $$	$$M_\odot $$	1.60
$m_2$	$M_\odot$	1.17
*M*	$M_\odot$	2.77
$s_\mathrm{z1}$	$\hbar$	$1.21\times 10^{57}$
$s_\mathrm{z2}$	$\hbar$	$4.73\times 10^{56}$
$d_\mathrm{so}$	$\mathrm{Mpc}$	40.00
$R_\mathrm{g}$	m	$4.11\times 10^3$
$R_\mathrm{in}$	$R_\mathrm{g}$	$2.00\times 10^5$
$\beta$		0.70
$e_0$		0.51
$R_\mathrm{min}$	$R_\mathrm{g}$	$0.65\times 10^5$
$R_\mathrm{av}$	$R_\mathrm{g}$	$1.14\times 10^5$
$R_\mathrm{max}$	$R_\mathrm{g}$	$2.00\times 10^5$
$v_1^\mathrm{max}$	*c*	$2.03 \times 10^{-3}$
$v_2^\mathrm{max}$	*c*	$2.79 \times 10^{-3}$
$\alpha _1$		$1.07 \times 10^{-4}$
$\alpha _2$		$7.81 \times 10^{-5}$
$\gamma$		$1.54 \times 10^{-5}$

**Fig. 1:**
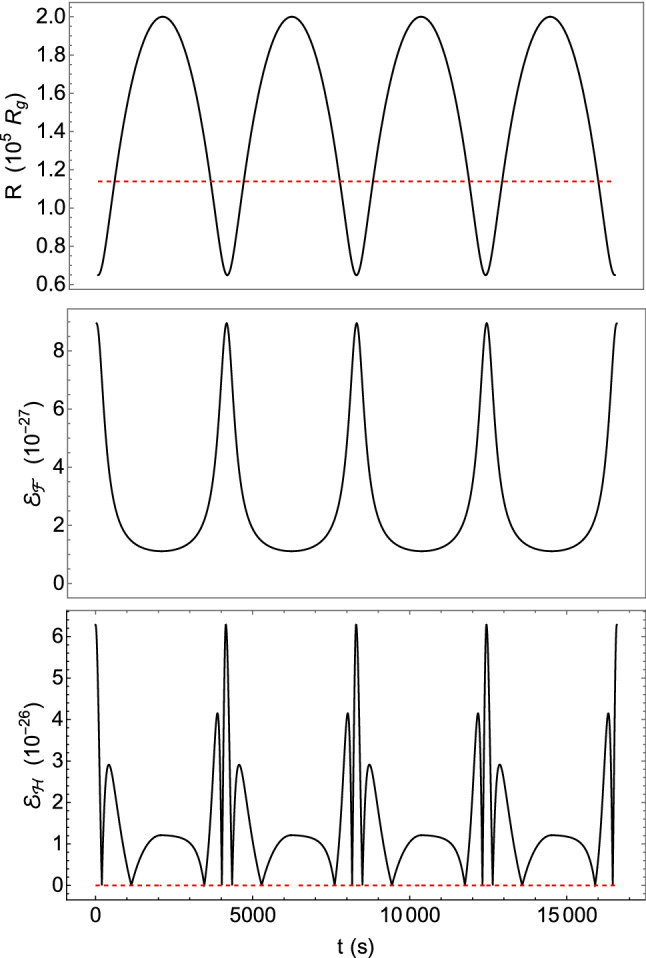
Plots of the functions *R*(*t*), $\mathcal {E}_{\mathcal {F}}(t)$, and $\mathcal {E}_{\mathscr {H}}(t)$. *Upper panel*: time evolution of the modulus of the relative radius (cf. Eq. (125)); the horizontal red dashed line corresponds to its average value (see Table 1). *Middle panel*: trend of $\mathcal {E}_{\mathcal {F}}(t)$ (see Eq. (138a)). *Lower panel*: behavior of $\mathcal {E}_{\mathscr {H}}(t)$ (cf. Eq. (138b)); the horizontal red dashed line represents the modulus of the mean EC contribution, which amounts to $9.31\times 10^{-53}$

The order of magnitude of the spin components $$s_\mathrm{z1}$$ and $$s_\mathrm{z2}$$ (in units of $$\hbar $$), physically representing the number of neutrons inside the NSs, is consistent with the values reported in the literature (which are of the order of $$ 10^{57}$$ neutrons) [107]; moreover, the magnitude of the parameters $$v_1^\mathrm{max},v_2^\mathrm{max},\alpha _1,\alpha _2,\gamma $$ confirms that the slow-motion, wide-separation, and weak-field hypotheses are fulfilled.

In order to estimate the EC contributions to the GR flux and waveform, we define (cf. Eqs. (129)–(133))

138a $$\begin{aligned} \mathcal {E}_{\mathcal {F}}(t)&\equiv \left| \frac{\mathcal {F}_\mathrm{EC}(t)}{\mathcal {F}_\mathrm{GR}(t)}\right| , \end{aligned}$$

138b $$\begin{aligned} \mathcal {E}_{\mathscr {H}} (t)&\equiv |\mathscr {H}^\mathrm{GR}_{11}(t)|-|\mathscr {H}^\mathrm{EC}_{11}(t)|. \end{aligned}$$

**Fig. 2:**
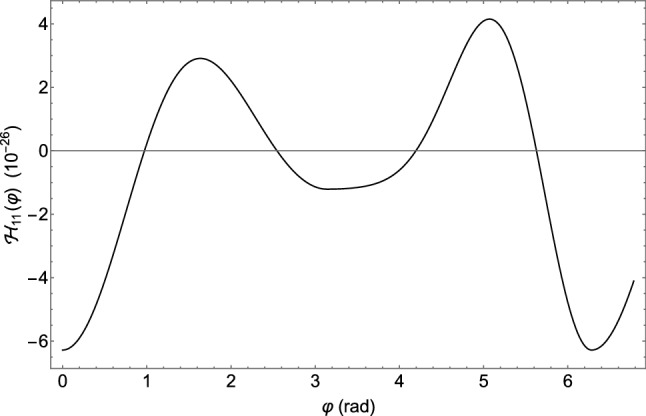
Gravitational waveform $\mathscr {H}_{11}$ as a function of the azimuthal angle $\varphi$. The $\varphi$-intercepts occurr at $\varphi =0.31\pi ,\ 0.81\pi ,\ 1.34\pi ,\ 1.79\pi$

The above quantities, along with the function *R*(*t*) representing the relative distance of the NSs, are shown in Fig. 1. From the plot of $\mathcal {E}_{\mathcal {F}}$, we see that the spin effects become more significant at the closest point of approach between the objects, where the gravitational field becomes more intense. This agrees with the spirit of EC theory, whose importance is expected to increase in the strong-gravity regime. In our example, the average contributions predicted by EC theory are smaller than GR ones by a factor of $10^{-23}$. This difference is consistent with the fact that the bodies are widely separated during their dynamical evolution. The EC corrections can be also figured out starting from the trend of $\mathcal {E}_{\mathscr {H}}$. As shown in Fig. 1, it goes from its minimum to its maximum values when the NSs get closer. Moreover, this function vanishes as soon as $\mathscr {H}_{11}(t)=0$ (cf. Eqs. (131) and (138b)). These points indicate when GR and EC effects become comparable and in our example we have $|\mathscr {H}_{11}^\mathrm{GR}| = |\mathscr {H}_{11}^\mathrm{EC}|\sim 10^{-52}$. The same information, expressed in terms of $\varphi$, can be inferred from the zeroes of $\mathscr {H}_{11}(\varphi )$, which occur at $\varphi \approx \frac{1}{4}\pi ,\frac{3}{4}\pi ,\frac{7}{4}\pi , \frac{11}{4}\pi$ (see Fig. 2; the functional form of $\mathscr {H}_{11}(\varphi )$ can be promptly deduced from Eqs. (131)–(133)).

## Conclusions

This work configures as a natural continuation of the research program started out in Ref. [66], where we have solved the GW generation problem in EC model at 1PN level by resorting to the Blanchet–Damour formalism. This general treatment finds an explicit application here, where the matter source is described by the Weyssenhoff fluid.

**Fig. 3:**
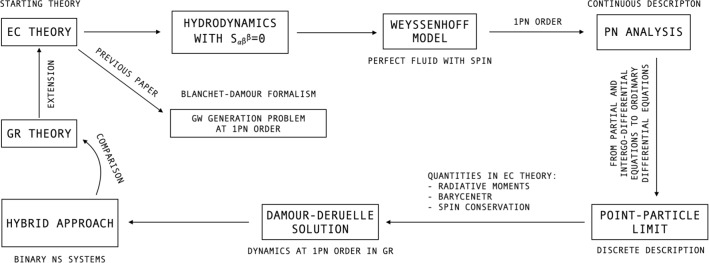
Flowchart of the paper. “Previous paper” refers to Ref. [66]

The structure of the paper is sketched in Fig. 3. In Sect. 2, we have summarized the key steps of the previous article. In this framework, the spinning PN source is supposed to be a generic hydrodynamical fluid system. For this reason, in Sect. 3.1, we have introduced the fundamental pillars of the EC hydrodynamics verifying the simplifying hypothesis that the torsion tensor has a vanishing trace, i.e., $S_{\alpha \beta }{}^\beta =0$. Subsequently, we have modeled the spin effects inside matter by employing the Weyssenhoff model of a semiclassical ideal spinning fluid supplemented by the Frenkel condition (see Sect. 3.2). The study of the Weyssenhoff fluid within the PN approximation scheme, representing a fundamental tool of the Blanchet–Damour formalism, is contained in Sect. 3.3. Both at 0PN and at 1PN level, the dynamics is ruled by a system of partial and integro-differential equations, whose resolution is extremely demanding. A less involved pattern can be obtained if we employ the point-particle procedure, which allows to characterize the fluid dynamics in terms of ordinary differential equations by going from a continuous picture to a discrete description of the system (see Sect. 4). We have then derived, within EC theory and for the particular case of binary systems, the 1PN formula (116) of the center of mass position and the general expressions (118)–(122) of the radiative multipole moments. Starting from these results and the conservation law (105) of the spin vector, we have resorted to the Damour–Deruelle solution in GR (which has been briefly discussed in Sect. 5.1) to set up a hybrid approach for dealing with binaries of spinning PN NSs, where we have provided some numerical estimates of the EC contributions to the flux and the waveform (see Sects. 5.2 and 5.3).

This paper contains some new theoretical results, which can be summarized as follows:

1. development of a general pattern (subject to the hypothesis $S_{\alpha \beta }{}^\beta =0$) for the hydrodynamics in EC theory, where the spin effects are modeled through the tensors $\varPhi ^{\alpha \beta }$ and $\tau _{\mu \nu }{}^\lambda$ (cf. Eq. (30));
2. PN investigation of the Weyssenhoff fluid, which predicts at 0PN level that: (*a*) the translational motion matches the Newtonian Euler equation (see Eq. (76b)); (*b*) the rotational dynamics reduces to a homogeneous continuity equation (see Eq. (76c)); (*c*) the expressions of the luminosity and the gravitational waveform reproduce formally the corresponding GR quadrupole formulas (cf. Eqs. (127) and (128));
3. derivation of the radiative multipole moments for the Weyssenhoff fluid both in the fine-grained and the coarse-grained (for an *N*-body and a binary system) descriptions, see Eqs. (88)–(92), (110)–(114), and (118)–(122);
4. computation, for the first time in the literature, of the numerical value of the NS spin as conceived in the Weyssenhoff semiclassical model (cf. Eq. (135));
5. hybrid scheme for providing a first estimate of the EC contributions to the GWs emitted by a binary NS system.

The calculation of the parameter *n* appearing in the spin formula (135) has led to established results for the number of neutrons inside a NS. Moreover, our investigation has revealed that EC contributions become more important when the gravitational field strength grows (see Fig. 1). Therefore, we could in principle extend our hybrid scheme to binary BH systems and evaluate the corrections to the radiated power and the asymptotic amplitude foretold by EC model. If we suppose that, similarly to Eq. (135), the BH spin can be written as

139 $$\begin{aligned} s= n\hbar \frac{4\pi }{3}\left( \frac{2Gm}{c^2}\right) ^3, \end{aligned}$$

(*m* being the BH mass) then we obtain that the EC effects are, for known astrophysical masses (i.e., $6 M_{\odot }\lesssim M \lesssim 10^{10} M_{\odot }$, with *M* the total mass of the system), between 23 and 13 orders of magnitude lower than GR ones. These differences are justified by the fact that our approach is restricted to compact binaries in their early inspiralling stage. Despite that, our analysis permits to infer that EC corrections can fulfill a relevant role in the later evolution phases. This topic might be useful for testing quantum phenomena in the strong-gravity regime, and it is worth examining it in a separate paper. Furthermore, GW phenomena could lead to interesting implications in the context of generalized EC theories. In particular, Ref. [108] puts forth a model where the gravitational Lagrangian has an additional quadratic-torsion term whose coupling constant differs from the Newtonian gravitational constant *G*. This new contribution changes the interaction strength between torsion and matter fields and hence novel results can be found also in the GW framework.

In the context of the GR pole-dipole approximation, the lowest-order “classical spin” contributions to the mass-type and current-type radiative moments emerge at 1.5PN and 0.5PN level, respectively [80, 110]. Therefore, in EC theory we recover *formally* the same expressions as in GR, see Eqs. (118)–(122). This is a consequence of the Frenkel condition (70) and the ensuing PN expansions of the tensors $T^{\mu \nu }$ and $\mathcal {S}^{\mu \nu }$ (see Eqs. (81) and (82)). If we define the Kerr angular momentum as $\mathcal {J}=\tfrac{Gm^2}{c}a$ with $a\in (0,1)$, we can provide an estimate of the effects introduced by EC theory on a single body by evaluating the ratio $s/\mathcal {J}$ by means of Eqs. (135) and (139). If we choose $a=0.5$, in the NS case we have $s/\mathcal {J}\sim 10^{-11}$ for $m\in [1.1,2.2]\ M_\odot$, while for a BH $s/\mathcal {J}\sim (10^{-12}-10^{-2})$ with $m\in [3,10^{11}]\ M_\odot$.

In conclusion, this paper prepares the ground for a systematic study of spinning PN binaries in EC theory. This entails a comprehensive investigation of the 1PN dynamics of binary systems in EC theory, which deserves consideration in a separate paper.

## Data Availability

This manuscript has no associated data or the data will not be deposited. [Authors’ comment: Data sharing not applicable to this article as no datasets were generated or analysed during the current study.]

## Appendix A: Post-Newtonian expansion of the Riemann tensor

We report selected PN expansions of the Riemann tensor components (see Eq. (8)). These are given by

A.1 $$\begin{aligned} {}^{(3)}R^k_{\ l0i}&= \partial _i\left( \frac{\chi }{2} c\,{}^{(1)}s_{kl}+\dfrac{4}{c^3}\partial _{[l}V_{k]}\right) \nonumber \\&\quad +\dfrac{2}{c^3}\delta _{i[k}\partial _{l]}\partial _t V, \end{aligned}$$

A.2 $$\begin{aligned} {}^{(2)}R^k_{\ lij}&= \dfrac{2}{c^2}\left( \delta _{k[j}\partial _{i]}\partial _l+\delta _{l[i}\partial _{j]}\partial _k\right) V, \end{aligned}$$

A.3 $$\begin{aligned} {}^{(2)}R^k_{\ 00i}&=\dfrac{1}{c^2} \partial _i \partial _{k}V, \end{aligned}$$

A.4 $$\begin{aligned} {}^{(4)}R^k_{\ 00i}&=-\chi \left[ \partial _i\left( {}^{(1)}s_{kp}v^p\right) +\frac{1}{2}\partial _t({}^{(1)}s_{ki})\right] \nonumber \\&\quad +\frac{1}{c^4}\delta _{ki}\left[ \partial _lV\partial _lV+\partial _t\partial _tV\right] \nonumber \\&\quad -\frac{1}{c^4}\left[ 4V\partial _k\partial _iV+3\partial _kV\partial _iV\right. \nonumber \\&\quad \left. +2\partial _t(\partial _kV_i+\partial _iV_k)\right] ,\end{aligned}$$

A.5 $$\begin{aligned} {}^{(3)}R^k_{\ 0ij}&= \chi \, c\partial _{[j} {}^{(1)}s_{k|i]}\nonumber \\&\quad +\frac{2}{c^3}\left( 2\partial _k \partial _{[i} V_{j]}+\delta _{k[j}\partial _{i]}\partial _tV\right) , \end{aligned}$$

where ${}^{(n)}R^{\mu }_{\ \nu \alpha \beta }\sim \left( \dfrac{\bar{v}}{c} \right) ^n\dfrac{1}{\bar{d}^2}$ and we have used the Frenkel condition (70). The above equations give contributions in Eq. (77).

## Footnotes

1 It is possible to show that Eqs. (6) and (7) are not independent (see Ref. [78] for further details).

2 We have used the *multi-index notation*, where *L* denotes the multi-index $i_1i_2\dots i_l$, hence $I_L=I_{i_1i_2\dots i_l}$ [11].

3 By defining the *expansion tensor* $\vartheta _{\alpha \beta } \equiv \mathcal {P}_\alpha ^\mu \mathcal {P}_\beta ^\nu \nabla _{(\nu } u_{\mu )}$, it is easy to prove that the *expansion scalar* $\vartheta \equiv \vartheta ^\alpha _{\ \alpha }$ assumes the same form as in GR [85], i.e., $\vartheta =2\hat{\nabla }_\alpha u^\alpha$. This quantity describes how the fluid volume changes during the motion.

4 The relative velocity vector has the same notation as the retarded potential *V* (cf. Eq. (61a)); however, the latter will not be considered in the following sections, and hence no confusion arises in using the same symbol for both quantities.
